# The Use of FTIR Spectra for Classifying Plant Items in a Vertebrate Herbivore’s Diet

**DOI:** 10.1007/s10886-026-01716-4

**Published:** 2026-05-11

**Authors:** Marcel Schäfer, Margit Zohmann-Neuberger, Jennifer Sorensen Forbey, Johannes Tintner-Olifiers, Angelika Hromatka, Erich Inselsbacher, Chloé Dépré, Ólafur Karl Nielsen, Ursula Nopp-Mayr

**Affiliations:** 1 Institute of Wildlife Biology and Game Management, Department of Ecosystem Management, Climate and Biodiversity, BOKU University, Gregor-Mendel-Strasse 33, Vienna, 1180 Austria; 2 Department of Biological Sciences, Boise State University, 1910 University Drive, Idaho, Boise, ID 83725-1515 USA; 3 Institute of Statistics, Department of Natural Sciences and Sustainable Resources, BOKU University, Peter Jordan-Strasse 82, Vienna, 1190 Austria; 4 Ernst & Young denkstatt GmbH, Hietzinger Hauptstrasse 28, Vienna, 1130 Austria; 5 Institute of Soil Research, Department of Ecosystem Management, Climate and Biodiversity, BOKU University, Peter Jordan-Strasse 82, Vienna, 1190 Austria; 6 Natural Science Institute of Iceland, Smiðjuvellir 28, 300, Akranes, Iceland; 7 LPO AuRA Drôme-Ardèche, 18 Pl. Genissieu, Chabeuil, 26120 France

**Keywords:** Chemical fingerprints, Fourier-transform infrared spectroscopy (FTIR), *Lagopus muta*Montin, Phytochemicals, Random Forest classification, Rock ptarmigan

## Abstract

Availability and quality of vegetation are critical factors influencing herbivore nutrition and population dynamics. Fourier-transform infrared spectroscopy (FTIR) offers a promising approach to analyze herbivore diets using spectral properties of phytochemicals to identify plant items. We evaluated the potential of FTIR to identify plant taxa and parts consumed by an herbivore species. Crop contents from 236 rock ptarmigan (*Lagopus muta* Montin) individuals from Iceland, collected over nine years, were separated into pure fractions of plant taxa and parts (e.g., berries, leaves) and analyzed using FTIR in the mid-IR region (4000 –400 cm⁻¹). We classified plant taxa and parts with PCA and Random Forests (RF) based on spectral signals. FTIR revealed distinct chemical fingerprints for plant taxa and parts, consistent with previously established variation in lipids, proteins, carbohydrates, and chemical defenses. RF yielded high classification accuracy for plant parts (96.7%) and moderate accuracy for taxa (85.5%), confirming the method’s reliability. FTIR overcomes limitations of traditional genetic analyses by identifying plant parts with varying nutritional quality within species. FTIR provided insights into biochemical properties of plant items but could not distinguish chemically similar items. Future research should expand spectral reference libraries combining FTIR with quantification of phytochemicals and DNA metabarcoding.

**Supplementary Information:**

The online version contains supplementary material available at 10.1007/s10886-026-01716-4.

## Introduction

Availability and quality of vegetation are critical biotic factors determining the nutritional status of herbivores with cascading effects scaling up to population levels (Thapa et al. 2021). These factors depend on biotic and abiotic drivers (Katoch 2023), where selection by herbivores relative to availability corresponds to specific plant features (Alm et al. 2002). Foraging decisions of herbivores are based on interacting determinants at multiple temporal and spatial scales, including nutritional needs, physiological tolerance to plant defenses (e.g., lignin, secondary metabolites), and trade-offs with other potential stressors like competition, predation, or weather conditions (Moss 1991; van Beest et al. 2010; McArthur et al. 2014). Understanding herbivore diet composition can provide insight into abiotic (climate) and biotic (species interactions) conditions experienced by herbivores (Hanley 1982). Since diet composition is linked to herbivore health and demographics (Brittas 1988; Degabriel et al. 2009) it can be translated into management strategies targeting vegetation availability or quality (Youngentob et al. 2025).

Various methods have been employed to address diet composition of vertebrate herbivores, each holding specific strengths and weaknesses. Traditional approaches such as microscopic analyses of intestinal contents or excreta (Kuc 1964; Zbinden 1984; Filacorda et al. 1997; Bertermann et al. 1998; Siano et al. 2011) rely on anatomical characteristics of shredded food items (e.g., (Swanson 1940; Kuc 1964; Eastman and Jenkins 1970; Picozzi et al. 1999). While feces can be collected non-invasively, digestion might bias which plants can be detected. Advanced techniques that focus exclusively on epidermal cells to identify recognizable fragments (‘‘recognition items’’) improve detection (Zettel 1974; Marti 1982, 1985), but are often restricted to relatively small sample sizes, single seasons or small geographical areas.

DNA metabarcoding is gaining use in dietary studies across taxa (e.g., Pegard et al. 2009; Sullins et al. 2018; Andriollo et al. 2019; Rytkönen et al. 2019; Sousa et al. 2019; Kartzinel and Pringle 2020).The main advantage of DNA metabarcoding is that it can provide a relatively accurate, high-resolution, and increasingly affordable approach, which can be used to provide a less biased picture of the diet of herbivores from fecal samples (Soininen et al. 2009; Sousa et al. 2019). Despite these advantages, DNA metabarcoding has limitations, such as the inability to determine specific plant parts or phytochemical composition, the need for advanced expertise in sample preparation and bioinformatics, reliance on a comprehensive plant genetics reference library, and potential bias from plant contamination (Ando et al. 2018).

In contrast, Fourier-transform infrared spectroscopy (FTIR) offers many advantages over the above-mentioned methods. This well-established technique analyzes chemical and physio-chemical properties of a wide range of biomaterials (e.g. Lopes et al. 2018; Doi et al. 2020) facilitating multiple applications in ecological research (Foley et al. 1998; White et al. 2025). Initially used to understand forage quality (Norris et al. 1976), FTIR can also be applied to fecal samples (Lyons and Stuth 1992), ingesta, or forage samples (Garnick et al. 2018) where “fecal-IR” is used to determine the nutritional status of free ranging ruminants (e.g., Tellado et al. 2015; Corlatti 2020). FTIR offers high molecular selectivity and when used to compare undigested plants’ spectral fingerprints to fecal samples it can provide an in vivo window into the physiological mechanisms mediating plant-herbivore interactions.

To date, fecal-IR largely focuses on diet composition in terms of crude protein content or digestibility (Dorgeloh et al. 1998; Kho et al. 2023). Only a few studies have demonstrated that FTIR can classify species and plant parts consumed by herbivores (Url et al. 2015; Nopp‑Mayr et al. 2020). However, testing the sensitivity and robustness of the FTIR method against plant parts that differ in chemical composition is a necessary first step to classify food items in fecal samples. Such testing should address if spectral signals of plant taxa and plant parts allow for their reliable identification. Testing should also be guided by herbivore foraging decisions. Fecal samples of free-ranging herbivores represent a “black box” of unknown phytochemical composition stemming from variation in both selection and digestion of plants by herbivores. As such, initial method validation should be based on the largest particles of plants selected by herbivores. Herbivorous grouse (Tetraoninae) species represent ideal taxa where this validation can be done. Within the crops of grouse, food items (i.e., plant taxa and parts) are present as comparatively large particles that allow for macroscopic identification and thus method verification. Analyzing crop contents also offers advantages over field-collected plant samples, as it accounts for herbivore food selection, which cannot be adequately anticipated by human sampling. Crop contents offer a unique opportunity to assess the capacity of FTIR to classify plants relative to the functional traits of plant taxa and plant part types selected by herbivores.

Here, we leverage data from a long-term study on an herbivorous grouse species in Iceland, to test and demonstrate both methodological strengths as well as potential limitations of the FTIR approach. Crop contents of 236 individual rock ptarmigan (*Lagopus muta* Montin) and previous macroscopic classification of plant items allowed for testing the discriminative power of FTIR in terms of plant taxa and parts. Assessing classification accuracy is a precondition for future studies that use non-invasively gained fecal samples of herbivores to monitor food selection.

We targeted the following research questions: (i) Do spectral signals of food plant taxa selected by rock ptarmigan allow for their identification?; (ii) Does FTIR support the identification of specific plant parts, which might otherwise not be determined via DNA metabarcoding?; (iii) Do the spectral signals provide a reliable basis for plant taxa and plant part classification that vary in phytochemistry?

## Methods and Materials

***Model Organism Rock Ptarmigan (Lagopus muta***). The rock ptarmigan is a circumpolar distributed grouse species of alpine and polar tundra regions. In Iceland, rock ptarmigan is the only breeding grouse species, inhabiting heath and grassland up to 800 m above sea level (a.s.l) (Nielsen et al. 2013; Dépré and Nielsen 2023). Rock ptarmigan are predominantly herbivorous with invertebrates only constituting an important part of the diet during the juvenile stage after hatching (Weeden 1969; Dépré and Nielsen 2023). Berries, woody shoots with buds, catkins, seeds, bulbils, and leaves are the most important plant parts for rock ptarmigan in Iceland in late summer and autumn when food supply is abundant. Plant species that usually dominate the birds’ autumn diet are *Betula nana*, *Empetrum nigrum*, *Dryas octopetala* and *Salix herbacea* (Dépré and Nielsen 2023). During the winter months, catkins and leaf buds gain importance (Weeden 1969; Garðarsson and Moss 1970). Iceland’s rock ptarmigan is an ideal model species for testing the FTIR for food item identification as it feeds on a relatively low number of plant taxa and parts. The crop samples from collected rock ptarmigan individuals offer a unique opportunity to separate and validate FTIR on food items selected by free-ranging rock ptarmigan.

***Study Area****.* The study area is located around Lake Mývatn in north-eastern Iceland mainly reaching from the coast up to 800 m a.s.l. with the highest peak reaching 1,200 m a.s.l. The study area is dominated by heath and meadow vegetation that includes *Betula nana* (dwarf birch), *Salix phylicifolia* (tea-leaved willow), many species from the heather family (Ericaceae, including *Empetrum nigrum*), grasses (*Poaceae*), sedges (*Carex* spp.), moss, and lichens. The climate is maritime with mean temperatures during the warmest month (July) ranging from 9.5 °C to 10.6 °C and mean temperatures during the coldest month (February) ranging from − 1 °C to −3.7 °C. The average annual precipitation at the study area during the collection period decreases from the coast (662 mm) to the inland (464 mm) (Snæþórsson 2012; Nielsen et al. 2013).

***Sample Collection.*** The crop samples (*n* = 236) used in this study originated from an earlier study, where rock ptarmigan individuals were collected to assess health parameters and population change (Guðmundsson 2015; Stenkewitz et al. 2016; Dépré and Nielsen 2023). The birds were collected using a shotgun within one week at the end of September and beginning of October of each year, covering a total of nine years (2006–2014). Recorded altitudes of the sampled individuals ranged from 0 to 759 m a.s.l. The collection of ptarmigan was authorized by the Natural Science Institute of Iceland under law 64/1994, Chap. 4, article 7.

***Sample Preparation – Macroscopy***. To derive pure plant fractions from the crop contents, the plant matter was separated according to species (or a higher taxonomic) level and plant parts using plant reference guides for Iceland (Löve 1983; Lid and Lid 1994; Kristinsson 2010). The definition of plant parts for vascular plants followed Garðarsson (1971), with the following groups occurring in our samples: (1) infructescences, (2) male catkins, (3) berries, (4), stems with buds, and (5) leaves. After separation, each plant sample was dried in an oven at 50 °C for five days.

***FTIR Analyses of Pure Plant Fractions.*** For FTIR analyses, the pure plant part samples yielded from macroscopic separation were dried in the oven at 80° C for 48 h. The dried samples were ground to a homogenous powder using a Retsch ^®^ vibrating ball mill (MM200). Samples with hard seeds or tough fiber were pre-ground with a mortar and pestle and then transferred into the ball mill. FTIR spectra were recorded on a Bruker ^®^ FTIR spectrometer (Tensor 27) in the Attenuated Total Reflectance (ATR) mode. Spectra were recorded in the mid-IR region (4000–400 cm^− 1^) at a spectral resolution of 4 cm^− 1^, collecting 32 scans. Five to ten replicates were taken per sample. Replicates were vector-normalized and averaged per sample with the integrated software OPUS ^®^ 8.5.

***Band Assignment***. For the band assignment the mean spectra of the plant taxa and plant parts were used. Peak search was carried out manually, supported by the OPUS ^®^ 8.5. single peak search. To allow a higher resolution of certain spectral regions second derivative spectra with nine smoothing points were created (Wang et al. 2008a). The band assignment was based on existing literature of FTIR spectra with a focus on wavenumbers representing plant cell wall components (e.g., celluloses, lignins) and phytochemistry (e.g., nutrients and chemical defenses) (Table 1, Supplemental Table S1).

**Table 1:** Band assignment of selected peaks of interest identified in spectra of plant items in crops of *Lagopus muta* that represent presumed phytochemical groups based on plausible molecular origin and literature. Plant taxa include: *Betula nana (Bn)*,* B. pubescens (Bp)*,* Empetrum nigrum (En)*,* Vaccinium = Vaccinium* spp. *(V)*,* Salix herbacea (Sh)*,* S. phylicifolia (Sp)*,* and Dryas octopetala (D).* Plant parts include: Catkins (C), infructescence (IFR), Berries (B), Stems with buds (S) and Leaves (L). > or < indicates the relative peak height compared to plant taxa or part listed. * indicates spectra that are distinct for a set of plant taxa or parts. PC1 and PC2 represent Principal Component analysis. RFp and RFtp represent Random Forest analysis for parts or plant taxa and parts, respectively

Presumed phytochemical group	Observed spectral pattern in plant taxa and parts	Plausible molecular origin	Evidence from statistical analysis	Wavenumber range in sample set (cm^− 1^)	Wavenumber range in literature (cm^− 1^)	Reference(s)o
Nutritional						
Carbohydrates	En > V; IFR > C	O-H stretching of hydroxyl groups	PC1 (-) PC2 (+)	3297 − 3289	3400 − 3000	Schwanninger et al. (2004)
Carbohydrates (sugars)	* Distinct in V and En	C–C–H vibrations of fructose	PC1 (-)	818 − 816	817	Max and Chapados (2007)
Carbohydrates (sugars)	* Distinct in V and En	C–C–H vibrations of fructose	PC1 (-)	776	778	Max and Chapados (2007)
Lipids (unsaturated fatty acids)	IFR > V > En *Missing in all other parts	=C-H stretching of cis alkenes in unsaturated fatty acids	PC2 (+)	3016 − 3010	3030–2990	Wang et al. (2008b)
Lipids (triglycerides)	IFR > V > En * Missing in C, S * Minor peak in D	C = O stretching in triglycerides	PC1 (-) PC2 (+) RFtp (taxa part): 1751 − 1741	1745	1745	Zimmermann and Kohler (2014;2015)
Protein	Sh > IFR > C, Sp, D; V > En	Protein Amide I: Mainly C = O stretching	PC2(+)	~ 1650	1709 − 1583	Bartošová et al. (2015)
Protein	Sh > IFR > C, Sp, D; V > En	Protein Amide II: Mainly N-H bending	PC1 (+) PC2 (+)	~ 1540	1580 − 1540	Smidt et al. (2008)
Nutritional/Structural						
Lipids	C > IFR > V > S, En, D	Asymmetric stretching of methylene (CH_2_) groups	PC2 (+) RFp (part): 2936–2912	2922 − 2918	3000–2800	Smidt et al. (2008)
Lipids	C > IFR > V > S, En, D	Symmetric stretching of methylene (CH_2_) groups	PC2 (+)	2852 − 2850	3000 − 2800	Smidt et al. (2008)
Lipids	CBp > CBn > IFR > V > En > S > D	C = O stretching in ungonjugated ketones, carbonyls and ester groups	RFp: 1730	1738 − 1731	1738 − 1709	Schwanninger et al. (2004)
Structural Defense						
Lignin (reduced digestibility) Silica	B < all other parts	OH-stretch (phenolic group in lignins) SiO-H (H-bonded)	PC1 (+) PC2 (-) RFp: 3585 − 3545 RFtp:3547 − 3533	3600 − 3500	3577–3568 3620, 3740–3600, 3490	Poletto et al. (2014), Vârban et al. (2021) Volkov et al. (2021), Proskurnin et al. (2023)
Lipids (structural components, possibly cutin)	CBp > CBn > all other samples	C = O…H in esters (i.e., in cutin) Ester groups (H-bonded)	PC2 (-)	1710	1713 1711	Heredia-Guerrero et al. (2014) Mazurek et al. (2013)
Lignins (reduced digestibility)	L > Sh > IFR, CBn > Cbp, Sp > V > En	Aromatic skeletal vibration of lignin	PC1 (+)	1518 − 1515	1515–1505	Schwanninger et al. (2004)
Fiber (reduced digestiblity)	En > V; Sp > D > Sh; IFR > C	C-O and C-C stretching in cellulose	PC1 (-)	1026 − 1025	1032 − 1021	Javier-Astete et al. (2021)
Fiber (reduced digestibility)	En > V; C < IFR < all other parts	C-OH stretching in cellulose	PC2 (-)	996	997	Bhagia et al. (2022)
Chemical Defense						
Oxalate	S > L > all other parts	Asymmetric COO^−^ stretching in oxalate	PC1 (+)	1620	1620	Tintner et al. (2018)
Oxalate	S > L > all other parts	Ca-Oxalate	PC2 (-)	780	782	Tintner et al. (2018)
Oxalate and tannins	S > L > all other parts	Ca-Oxalate Hydrolysable tannins	PC1 (+) PC2 (-)	1315	1320 1325 − 1317	Tintner et al. (2018) Falcão and Araújo (2013)
Tannins	* Distinct in S	Hydrolysable tannins	PC2 (+)	1720	1731 − 1704	Falcão and Araújo (2013)
Tannins	* Distinct in S	(Hydrolysable) gallotannins	PC2 (-)	763	763 − 758	Falcão and Araújo (2013)
Phenolics	Sh > Sp > L, IFR > C > V > En	C-C aromatic vibration of phenolic compounds including: Sporopollenin Lignins Tannins	PC1 (+) PC2 (-) RFp: 1607	1608–1605	1606 1605 1600 1615 − 1606	Heredia-Guerrero et al. (2014) Zimmermann and Kohler (2014) Horikawa et al. (2019) Falcão and Araújo (2013)

***Statistical Analysis***. To assess if spectral signals of food plants selected by rock ptarmigan can discriminate among plant taxa and plant parts, we ran a Principal Component Analysis (PCA) using the ChemoSpec package (version 6.3.1, Hanson 2024) in R 4.5.2 (R Core Team 2025). The spectral region from 2500 cm^− 1^ to 1800 cm^− 1^ was removed prior to analysis as it contains the interfering signals of atmospheric CO_2_ (Ahmed et al. 2024) and the ATR crystal (Gupta et al. 2015) and does not provide signals of any relevant functional group.

We applied Random Forest classification (RF) algorithms (Breiman 2001; Liaw and Wiener 2002; Cutler et al. 2007) to test the discriminatory power of spectral signals at plant taxa and plant part level. RF were built using the packages randomForest (version 4.7–1.2, Liaw and Wiener 2002) and caret (version 7.0–1, Kuhn 2008) in R 4.5.2 (R Core Team 2025). The dataset was split into 80% training and 20% test data. To increase interpretability and reduce redundancy in the predictors, the variable importance (mean decrease in accuracy) from the initial Random Forest models was combined with our band assignment (Table 1) and literature research, to define groups of band aggregates (Boseley et al. 2024; do Prado Puglia et al. 2026). Models applied on the aggregated bands achieved slightly improved classification results and were therefore used for further analysis. Absolute peak height per band aggregation yielded the best classification. Defined groups of band aggregates are given in Table S11. Hyperparameter optimization included *mtry* (number of randomly selected variables at each split), *ntree* (number of decision trees in the RF), *maxnodes* (maximum number of end nodes per decision tree) and *nodesize* (minimum number of observations per end node) (Probst et al. 2019). *Mtry* was tested for values of 5, 10 and 20, *ntree* for 100, 300 and 500, *maxnodes* for 50 and 100 and *nodesize* for 1, 5 and 10. Optimal hyperparameters were determined via 10-fold cross-validation on the training data (the final parameters are given in Table S2 and S12). Variable importance was determined by calculation of the permutation importance (mean decrease in accuracy).

Plots were created using the R package ggplot2 (version 4.0.2, Wickham 2016). The displayed spectra were printed via OPUS ^®^ 8.5. SpectraGryph 1.2.17 was used for format editing. The software GIMP 2.10.38 (GIMP Development Team 2025) was used for figure editing.

## Results

***Ptarmigan Crop Samples.*** Among the sampled ptarmigan individuals (n_total_ = 236), 51 specimens were adults (22 females, 29 males) and 174 were juveniles (89 females, 85 males). Every year included at least one member of each age and sex class except for 2012, where no adult female individuals were sampled.

***Plant Taxa and Plant Part Identification – Macro-Histology.*** From the set of individual crops (*n* = 236), subsamples of distinct (pure) plant fractions were produced and assigned to taxonomic and plant part classes. Overall, 296 pure fractions of plant taxa or parts could be distinguished. Within these pure fractions yielded from the macro-histological separation and determination procedure, the following plant taxa and plant parts were detected: Reproductive parts of *Betula nana* L. (both infructescence and catkins) and *B. pubescens* EHRH. (only catkins); Berries from *Vaccinium* spp. L. (including both *V. uliginosum* or *V. myrtillus* which are morphologically indistinguishable) and *Empetrum nigrum* L.; stems with buds from *Salix herbacea* L. and from *S. phylicifolia* L.; leaves from *Dryas octopetala* L.

In terms of their occurrence (i.e., the number of sampled rock ptarmigan specimens, where plant taxa were detected), *Vaccinium* spp., *B. nana*, *D. octopetala*, and *E. nigrum* were the most abundant plant taxa detected in the crops of ptarmigan (Table S3). In terms of their proportional biomass in the diet of the sampled birds (i.e., mean relative biomass in %), *Vaccinium* spp., *E. nigrum*, and *D. octopetala* reached the highest proportion in rock ptarmigan diet across all years. However, nearly all plant species (except for *S. phylicifolia* and *B. nana*) occurred as predominant plant food component in single years or in individual ptarmigan (Table S3, see maximum values).

The predominant five plant parts detected were infructescence, catkins, berries, stems with buds, and leaves. Berries showed the highest occurrence within the sampled birds, followed by leaves and infructescence (Table S4). In terms of proportional biomass (mean or median values), stems with buds, berries, and catkins reached the highest proportions. Apart from infructescence, all plant parts predominated in a single year or in individual ptarmigan (Table S4, maximum values, Figure S1).

### FTIR Analyses

**Fig. 1:**
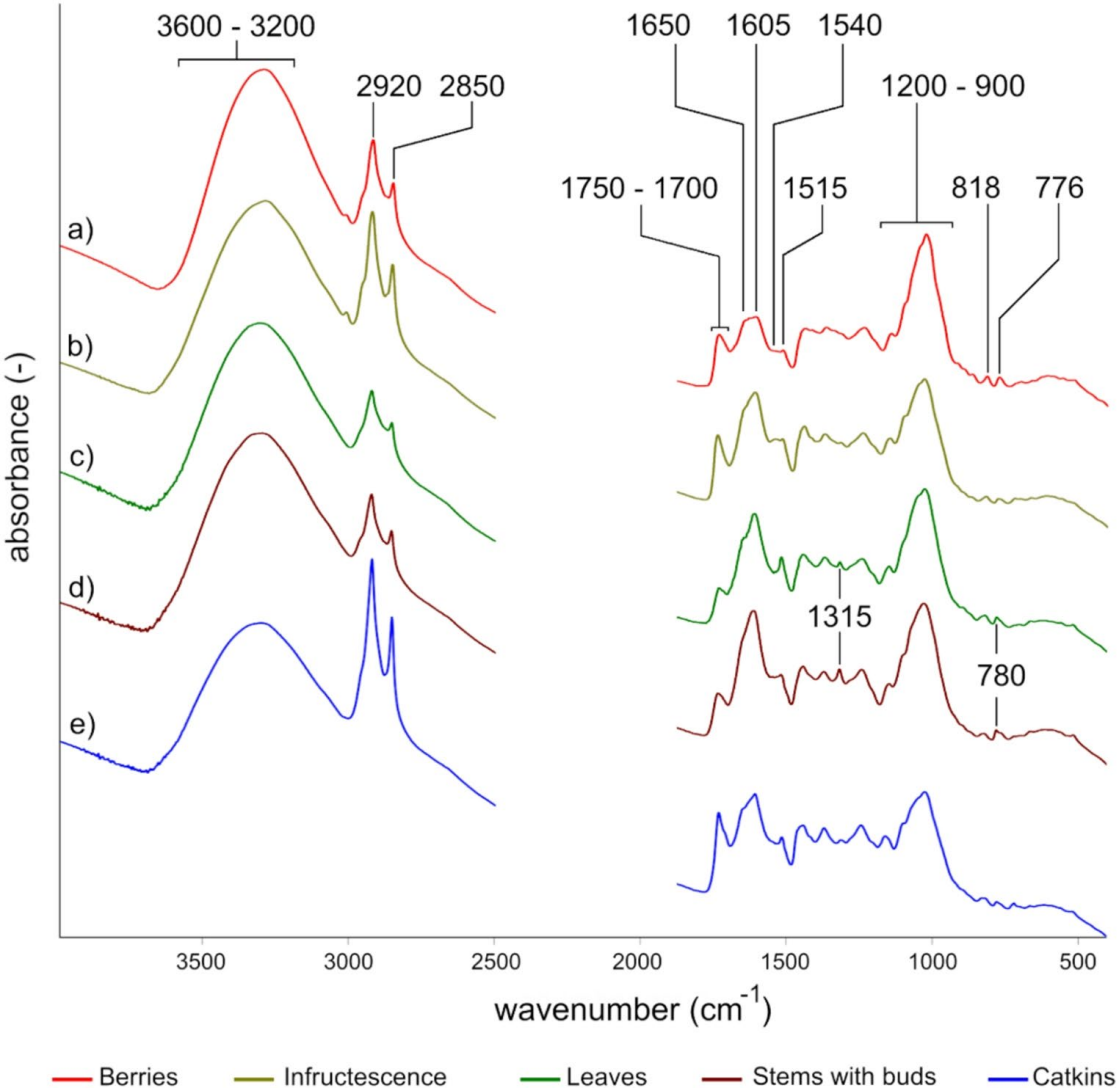
Average FTIR spectra plant parts representing reproductive parts (infructescence, catkins, and berries) and vegetative parts (stems with buds and leaves) found in the crops of individual rock ptarmigan. Vector-normalized spectra are shown for selected spectral regions. The region of CO_2_ absorption and crystal interaction (2500–1800 cm^−1^) was removed prior to analysis. Beams indicate distinct peaks and display the corresponding wavenumbers. For band assignments see Table 1. Spectra are shifted along the y-axis for better representation

*Spectral Features of Phytochemicals*,* Plant Taxa*,* and Plant Parts.* The analysis of FTIR spectra of crop samples indicated distinctive spectral features for plant taxa and parts within taxa (Fig. 1). The spectral features were first used to identify phytochemical fingerprints representing three key nutritional groups that included presumed carbohydrates, lipids, and protein, and two groups of plant defenses that included presumed structural and chemical defenses (Table 1). The carbohydrate fingerprint for our samples included increased peak heights at wavenumbers in the regions of 3300–3289 cm^−1^ indicating hydroxyl groups (OH-stretching) and distinct peaks at ~ 818 cm^−1^ and ~ 776 cm^−1^ indicative of fructose. The two lipid fingerprints included nutritional and structural lipids. Increased peak heights at ~ 2920 cm^−1^ and ~ 2850 cm^−1^ (CH_2_ stretching) indicating relatively high total hydrocarbon concentrations were attributed to both lipid fingerprints. The nutritional lipid fingerprint was additionally represented by increased peak heights at ~ 3013 cm^−1^ indicating unsaturated fatty acids that were paired with a peak maximum at ~ 1738 cm^−1^ within the 1750–1700 cm^−1^ carbonyl ester region and band broadening at ~ 1745 cm^−1^ indicative of triglycerides, including dietary lipids. The structural lipid fingerprint was represented by increased peak heights at ~ 1731 cm^−1^ within the 1750–1700 cm^−1^ carbonyl ester region and increased peak heights at ~ 1710 cm^−1^ indicative of cutin. The protein fingerprint included increased peak heights at ~ 1650 cm^−1^ and ~ 1540 cm^−1^. The structural defense fingerprint included wavenumbers indicating lignin or silica (~ 3600–3500 cm^−1^), cellulose (~ 1025 cm^−1^ and ~ 996 cm^−1^), lignin (~ 1515 cm^−1^), and cutin (described above). The chemical defense fingerprint included wavenumbers indicating oxalates (~ 1620 cm^−1^ and ~ 780 cm^−1^), oxalates and tannins (~ 1315 cm^−1^), tannins (~ 1720 cm^−1^ and ~ 763 cm^−1^), and phenolic compounds (~ 1605 cm^−1^).

**Fig. 2:**
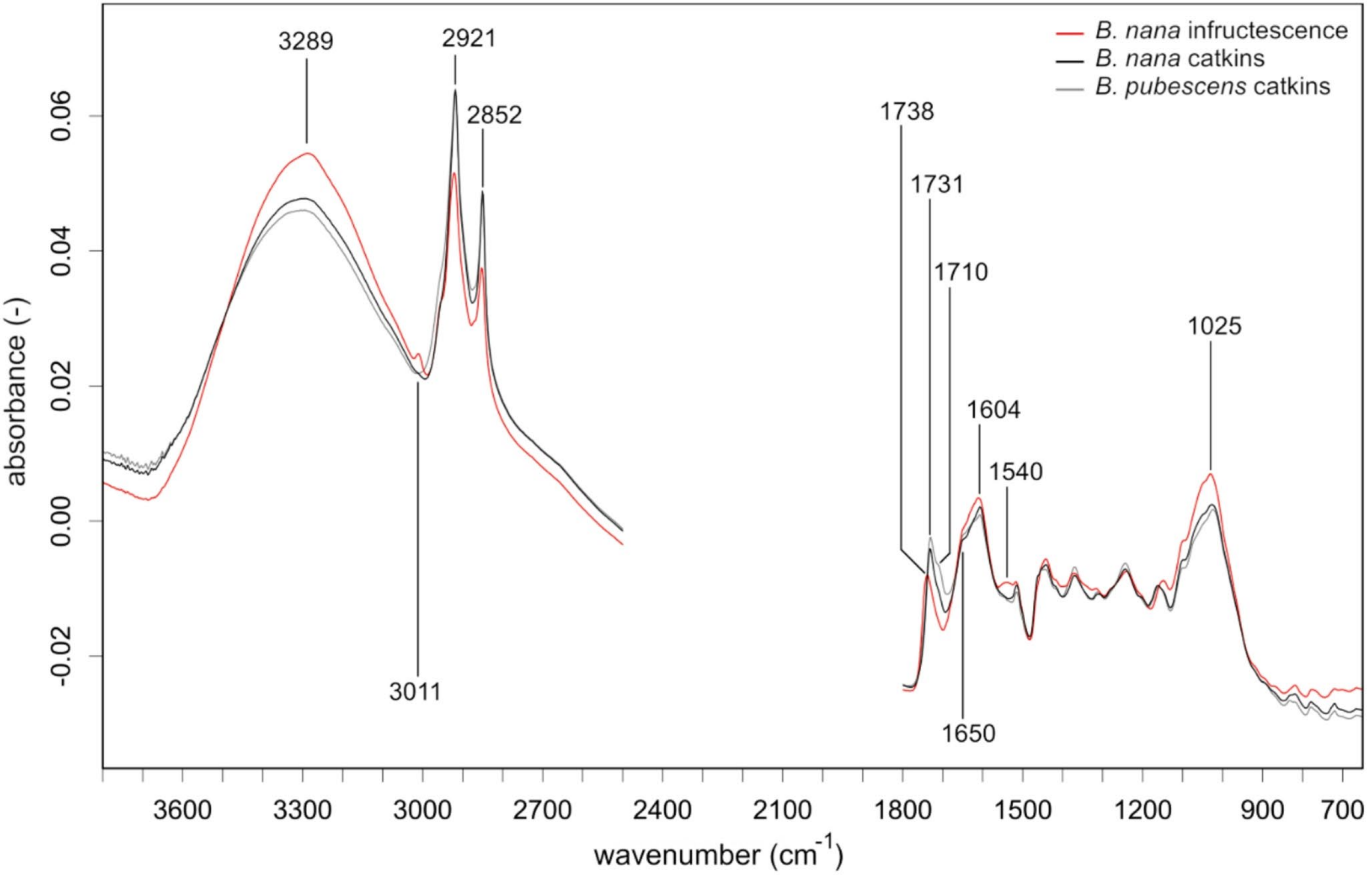
Average FTIR spectra plant parts of the two *Betula* species, found in the crops of sampled rock ptarmigan individuals. Vector-normalized spectra are shown for selected spectral regions. The region of CO_2_ absorption and crystal interaction (2500–1800 cm^−1^) was removed prior to analysis. For band assignments see Table 1

**Fig. 3:**
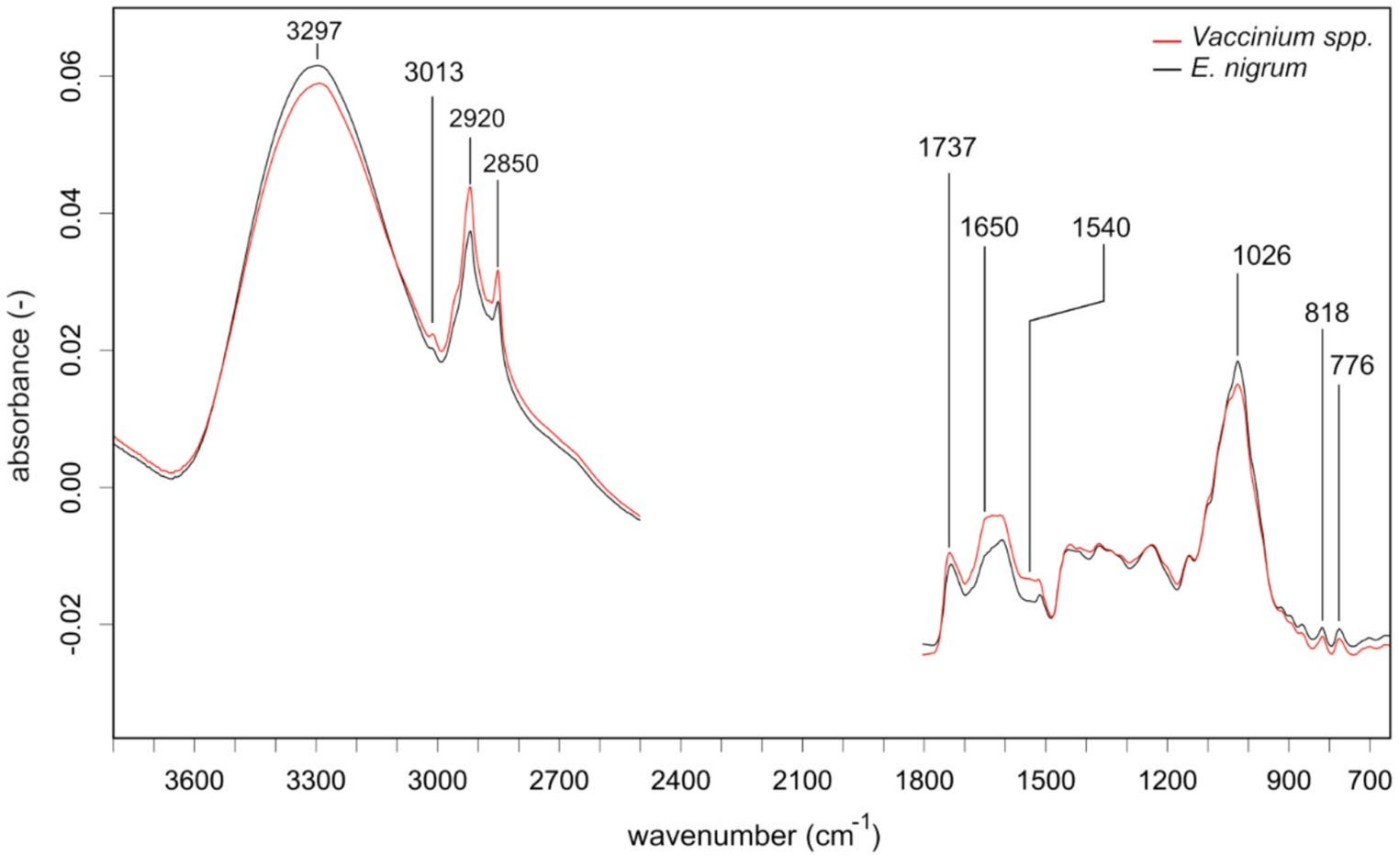
Average FTIR spectra of berries of *Vaccinium* spp. and *E. nigrum*, found in the crops of sampled rock ptarmigan individuals. Vector-normalized mean spectra are shown for selected spectral regions. The region of CO_2_ absorption and crystal interaction (2500–1800 cm^−1^) was removed prior to analysis. For band assignments see Table 1

**Fig. 4:**
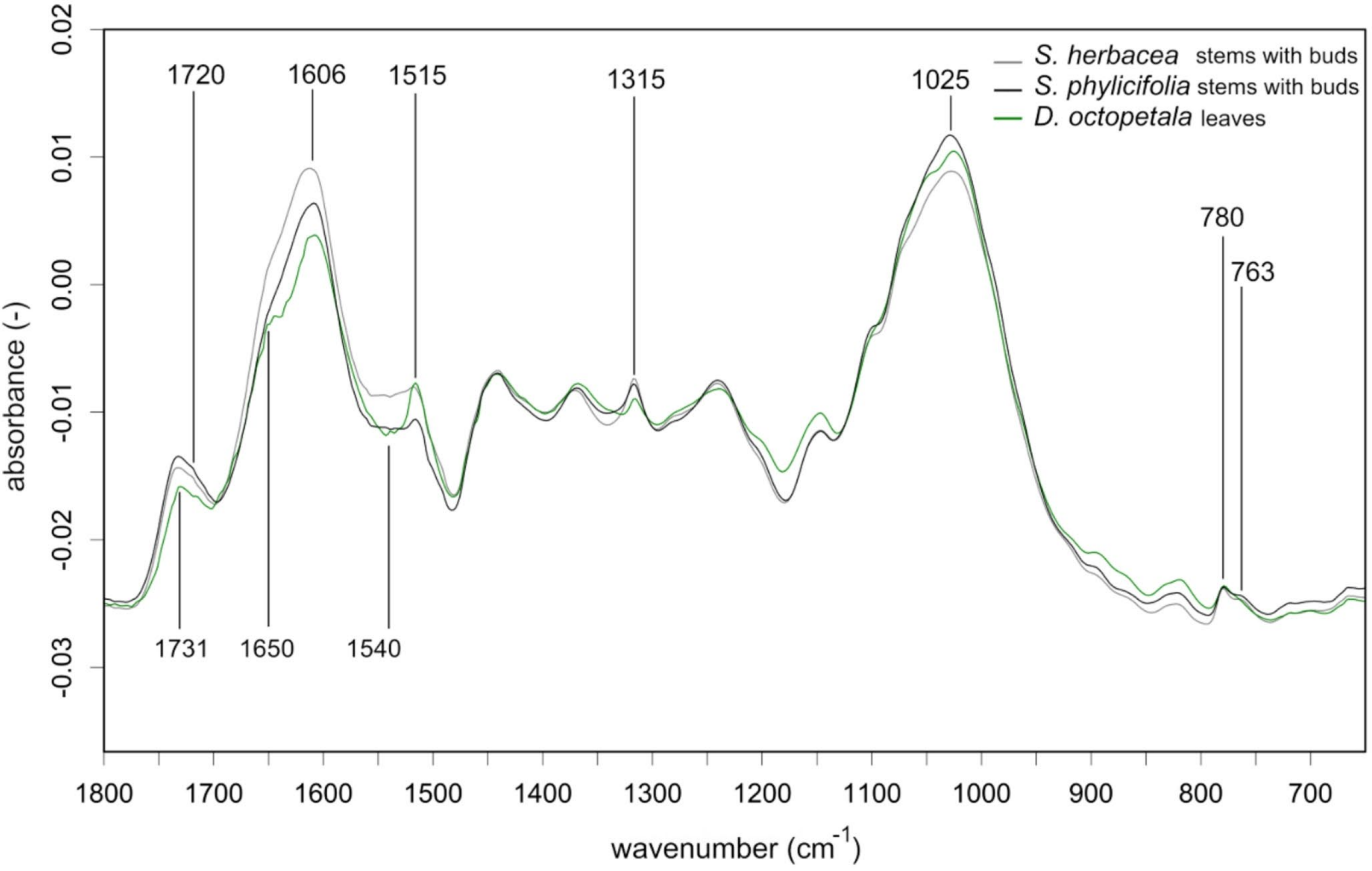
Fingerprint region of the average FTIR spectra of stems with buds of the two *Salix* species compared to signals of leaves of *D. octopetala*, found in the crops of sampled rock ptarmigan individuals. Vector-normalized spectra are shown for selected spectral regions. For band assignments see Table 1

*Signals within Spectral Clusters.* We also identified three spectral plant clusters that allow for further visual separation between plant parts within taxa or between taxa for a given plant part. The first spectral cluster represented reproductive plant parts that are predominantly wind dispersed and included catkins and infructescence of *Betula* species (Fig. 2). The second spectral cluster represented reproductive plant parts that are predominantly animal dispersed and included the berries of *E. nigrum* and *Vaccinium* spp. (Fig. 3). The third spectral cluster represented relatively permanent vegetative parts providing support and photosynthetic function that included the stems with buds of *S. phylicifolia* and *S. herbacea* and leaves of *D. octopetala* (Fig. 4). The spectral patterns were used to identify peaks of interest defined as those there were (1) distinctly present or missing within a plant part or taxa or (2) separated plant parts or taxa in the PCA or RF analysis and represented spectra with a plausible molecular origin (Table 1). The specific wavenumbers of peak maxima for the peaks or bands of interest shifted based on the set of samples investigated but remain within a range of wavelengths representing plausible origins for phytochemical traits (Table 1).

**Fig. 5:**
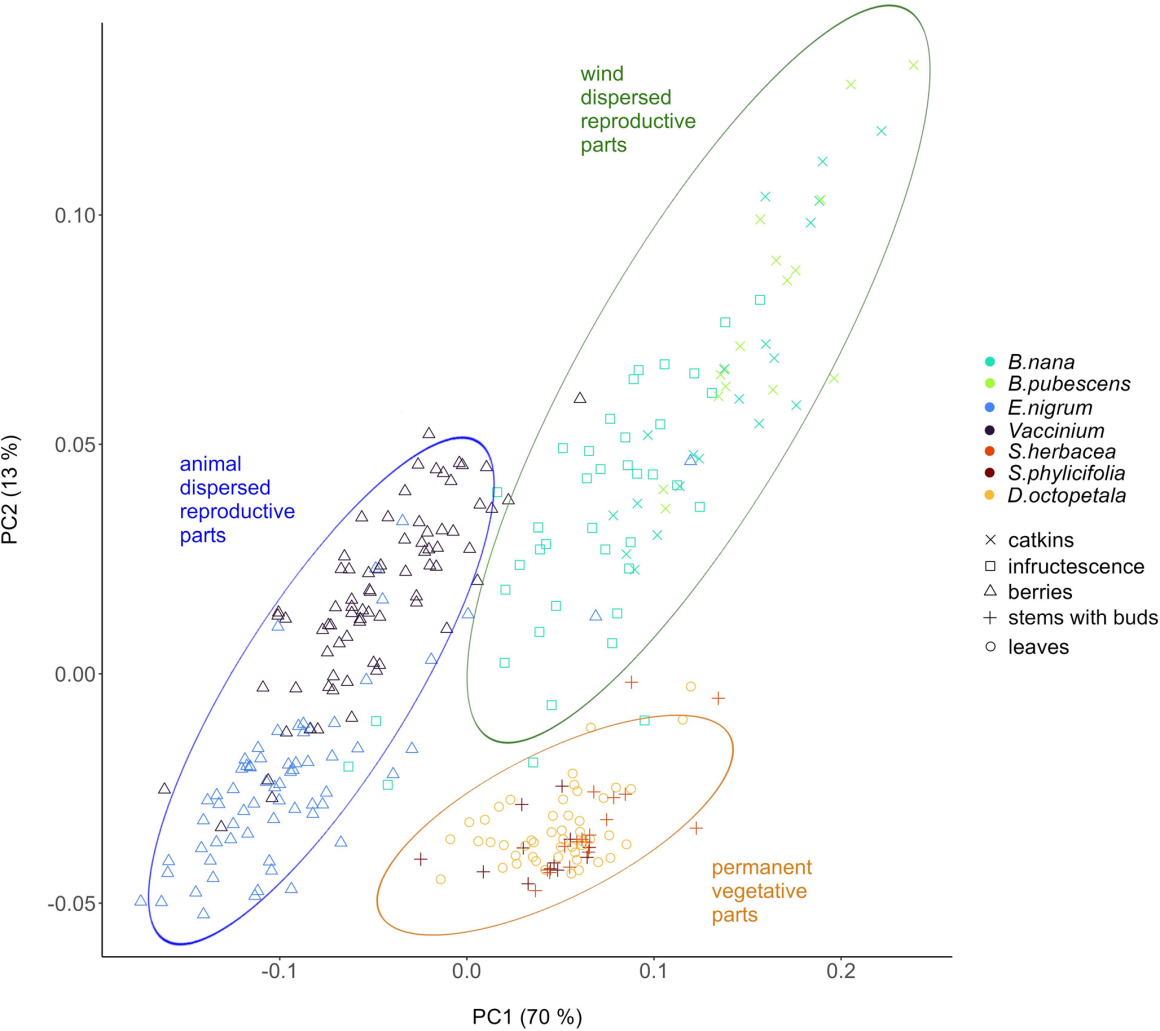
FTIR spectra separating (even closely related) plant species as well as plant parts within the same species. PCA scores plot of first (PC1) and second (PC2) principal component. Percentage in parentheses displays the explained variance of each principal component. Shapes indicate plant parts, symbol colors indicate plant taxa (*B. nana = Betula nana*,* B. pubescens = Betula pubescens*,* E. nigrum = Empetrum nigrum*,* Vaccinium = Vaccinium* spp. including both *V. uliginosum and V. myrtillus*,* S. herbacea = Salix herbacea*,* S. phylicifolia = Salix phylicifolia*,* D. octopetala = Dryas octopetala)*

***Principal Component Analysis.*** PCA of the spectra (excluding the wavenumber region between 2500 and 1800 cm^−1^) generally yielded a distinct separation of plant taxa and plant parts with principal components one and two explaining 70% and 13% of variance, respectively (Fig. 5). After the first and second principal component, no additional separation of groups was apparent. The first principal component showed separation of the two types of reproductive parts with berries of *Vaccinium* spp. and *E. nigrum* (negative scores) separated from catkins and infructescence of *Betula* (positive scores) with the permanent vegetative parts of *Salix* spp. (stems with buds) and *D. octopetala* (leaves) intermediate. Furthermore, catkins showed stronger negative scores than infructescence within *Betula* species along the PC1 axis. The second principal component primarily separated *Betula* species (positive scores) from *Salix* spp. and *D. octopetala* (negative scores). The PC2 also separated berries of *Vaccinium* spp. (positive scores) from berries of *E. nigrum* (negative scores) and showed some additional separation between catkins (positive scores) and infructescence (negative scores) within *Betula* species.

**Fig. 6:**
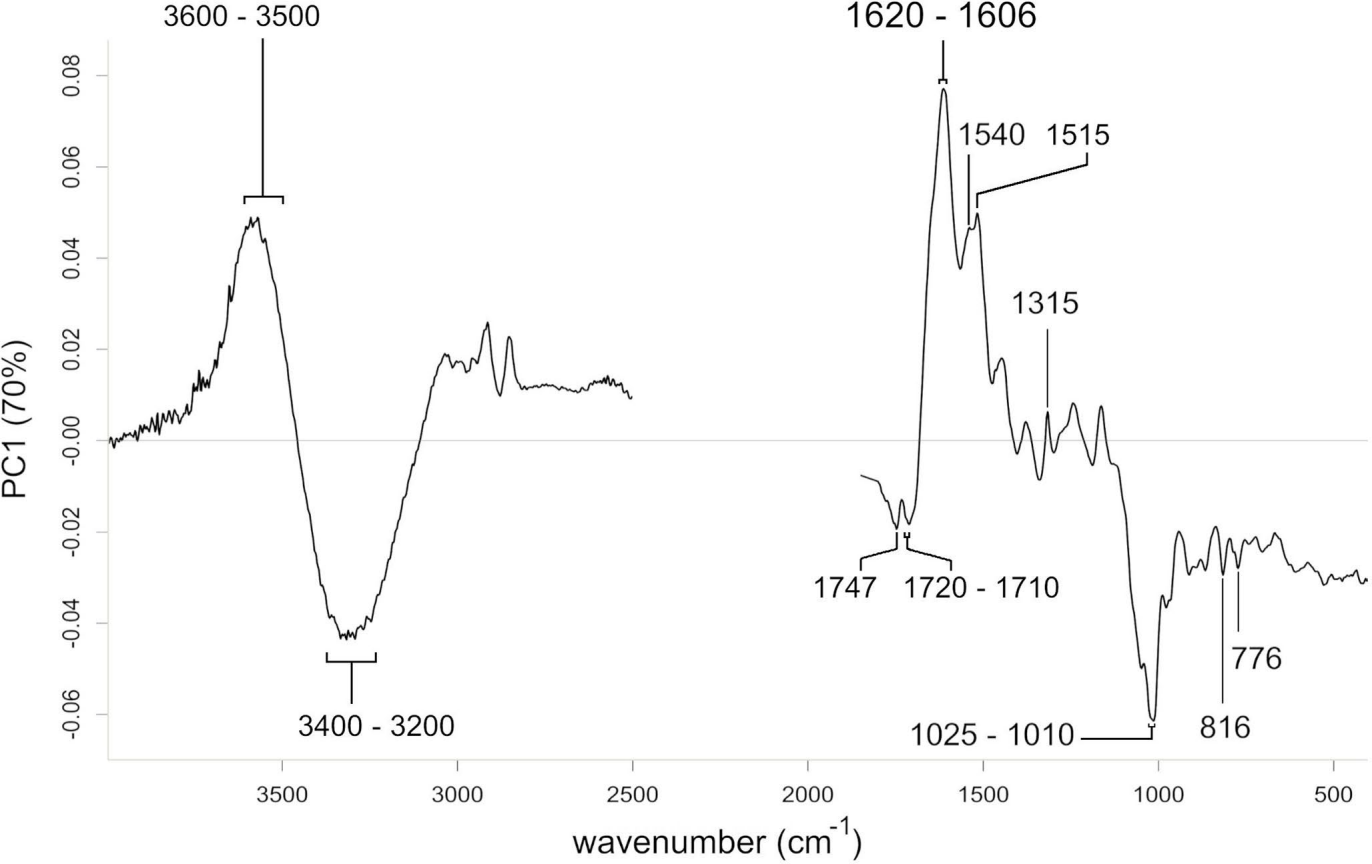
PCA loadings plot of the first principal component. Beams display wavenumbers (cm^−1^) with distinct positive or negative loadings. The region of CO_2_ absorption and crystal interaction (2500–1800 cm^−1^) was removed prior to analysis. For band assignment see Table 1

**Fig. 7:**
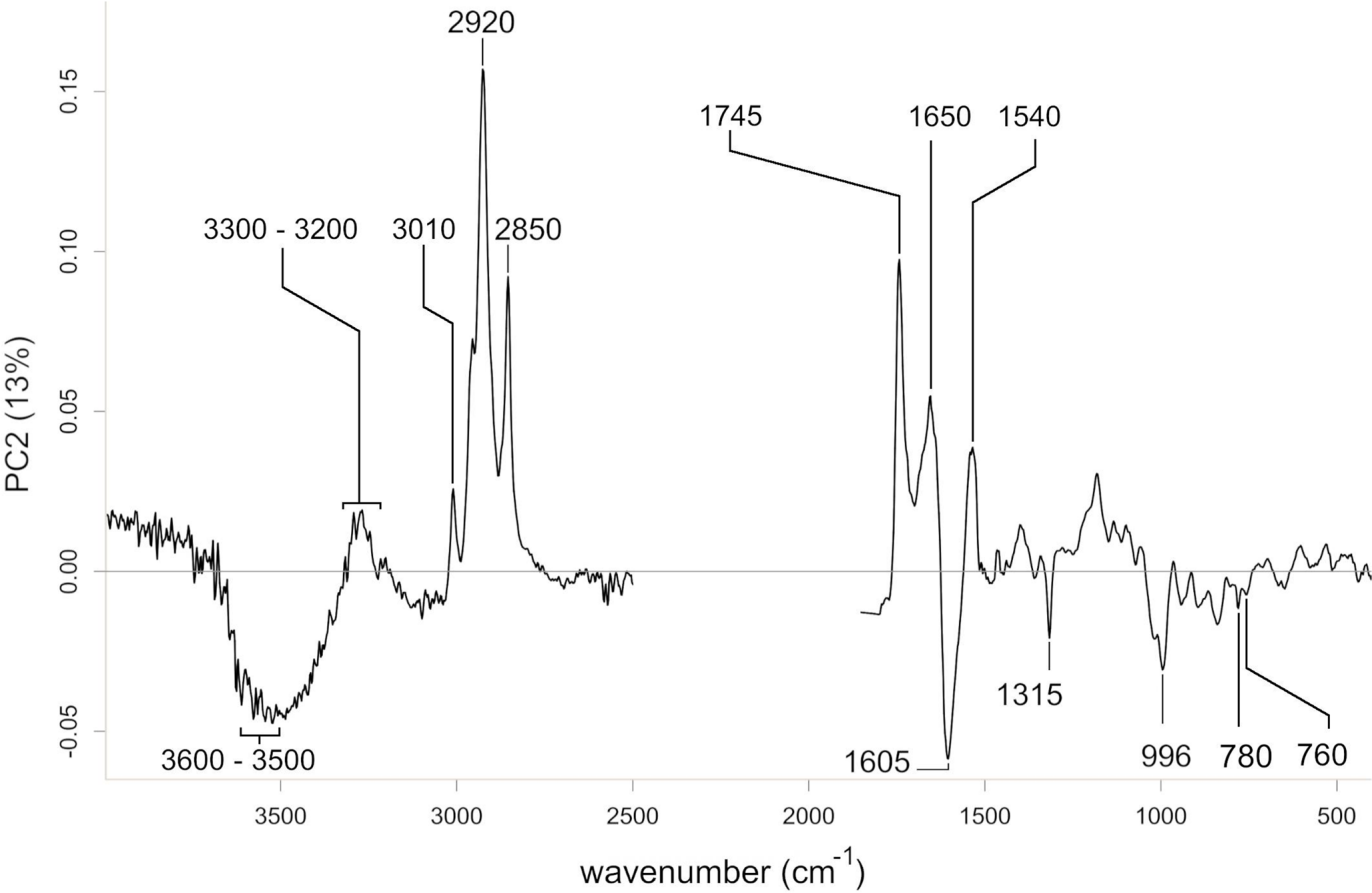
PCA loadings plot of the second principal component. Beams display wavenumbers (cm-1) with distinct positive or negative loadings. The region of CO_2_ absorption and crystal interaction (2500–1800 cm-1) was removed prior to analysis. For band assignment see Table 1

*Loadings*. The loadings of the first principal component suggested an increased absorbance at 3600–3500 cm^− 1^, 1620–1606 cm^− 1^, ~ 1540 cm^− 1^, ~ 1515 cm^− 1^, and ~ 1315 cm^− 1^ towards the positive PC1 scores (Fig. 6). The intensities of absorbance at ~ 3300 cm^− 1^, ~ 1747 cm^− 1^, 1720–1710 cm^− 1^, 1025–1010 cm^− 1^, ~ 816 cm^− 1^, and ~ 776 cm^− 1^ increase towards the negative PC1 scores. The loadings of the second principal component suggested an increasing absorbance at ~ 3010 cm^− 1^, ~ 2920 cm^− 1^, ~ 2850 cm^− 1^, ~ 1745 cm^− 1^, ~ 1650 cm^− 1^ and ~ 1535 cm^− 1^ towards the positive PC2 scores (Fig. 7). The absorbance at 3600–3500 cm^− 1^, 1605 cm^− 1^, 1315 cm^− 1^, 996 cm^− 1^, 780 cm^−^1, and ~ 760 cm^− 1^ increase towards the negative PC2 scores.

***Discriminative Power of Spectral Signals.*** The Random Forest (RF) algorithm identified the best discriminating wavenumbers or regions that might classify plant part or taxa. All band aggregates, except for the lipid absorbance at ~ 1710 cm^− 1^, appeared in varying order in the RF variable importance output of both RF models (Table S15, Table S18). Overall, plant parts were classified at a higher accuracy than taxa.

*Plant Parts.* The RF model for classification of plant parts (RFp) yielded an out-of-bag error of 2.98%. The prediction applied on the test data yielded an overall accuracy of 0.967 (95% CI: 0.887, 0.996) with a kappa-value of 0.953. The RFp model predicted catkins, berries, stems with buds and leaves with the highest accuracy (> 96%). The least accurate prediction was for infructescence (~ 88%). Further details on model performance (i.e., confusion matrix, statistics by class and variable importance) are given in Tables S13 to S15.

*Plant Taxa and Part.* The Random Forest model for classification of plant taxa and parts (RFtp) yielded and out-of-bag error of 11.11%. The prediction applied on the test data yielded an overall accuracy of 0.855 (95% CI: 0.742, 0.931) with a kappa-value of 0.826. The RFtp model predicted leaves of *D. octopetala* with the highest accuracy (100%), followed by *B. nana* infructescence, *Vaccinium spp.* and *E. nigrum* berries (> 90%), In classifying *B. nana* and *B. pubescens* catkins and *S. herbacea* stems with buds RFtp achieved an accuracy of ~ 87%. The least accurate prediction of taxa and part was shown for stems with buds of *S. phylicifolia* (~ 82%). Further details on model performance (i.e., confusion matrix, statistics by class and variable importance) are given in Tables S16 to S18.

## Discussion

Overall, we demonstrate that FTIR has the discriminative power to reliably classify plant taxa and parts selected as food by Icelandic rock ptarmigan. Spectra were more distinct among functional parts of plants than in different taxa sharing the same plant part. Results demonstrate the unique potential for FTIR to classify the forage selected by herbivores that may be based on distinct phytochemical traits rather than taxonomy.

PCA loadings were largely consistent with spectral distinction among plant parts. Lipid content appeared to increase towards the positive PC2 scores (2920 cm^−1^, 2850 cm^−1^). A positive PC2 loading at 1743 cm^−1^ and a negative PC1 loading at 1745 cm^−1^ indicated the increased contribution of triglycerides in the infructescence, *Vaccinium* berries, and to a lower extent the *E. nigrum* berries. The PCA loadings also indicated that signals of carbohydrates (~ 3300 cm^−1^, 1025 cm^−1^) increased in berries towards the negative PC1 scores and decreased in catkins, infructescence, stems with buds, and leaves towards the positive PC1 scores. The two signals for fructose (~ 818 cm^−1^ and ~ 774 cm^−1^) separated berries towards the negative PC1 axis indicating increased monosaccharide contents in the berries. A positive PC1 and negative PC2 loading at 1315 cm^−1^ indicated the separation of leaves and stems with buds from the remaining samples which was likely influenced by increased content of oxalate. Regarding plant species, only the berries of *E. nigrum* and *Vaccinium* exhibited a separation in the PCA. The positive PC2 loadings at ~ 1745 cm^−1^ indicated this separation to be based on increased contents of triglyceride lipids in the berries of *Vaccinium* spp. compared to *E. nigrum*. In addition, the PC1 scores for protein (~ 1650 cm^−1^, ~ 1540 cm^−1^) appeared to be lowest in *E. nigrum* berries which separated them towards the negative axis away from the *Vaccinium* berries and the remaining samples. A negative PC2 loading at ~ 995 cm^−1^, attributed to cellulose, indicated increased fiber concentrations in *E. nigrum* compared to *Vaccinium*.

The Random Forest models exceeded the separation of groups observed in the PCA and confirmed the capability of FTIR spectra to enable a credible assignment of plant parts even with a small sample size. The RF model for plant parts (RFp) yielded excellent classification performance, RFp provided the greatest improvement of the separations observed in the PCA by successfully classifying catkins distinct from infructescence and classifying leaves distinct from stems with buds. The RF model for plant taxa and parts (RFtp) performed less precise than the model for plant parts, however still achieved an accuracy of 0.855. As observed in RFp, RFtp was able to correctly classify *D. octopetala* leaves. The RFtp could not consistently classify different species with the same plant part except for berries. However, RFtp was successful in classifying *B. nana* catkins from other plant taxa and parts, but catkins of *B. pubescens* were partly misclassified as catkins from *B. nana* and vice versa. RFtp was also successful in classifying *Salix* stems and buds from other plant taxa and parts, but *S. phylicifolia* samples were partly misclassified as *S. herbacea* and vice versa.

In the RFp, the oxalate absorbance at 1620–1618 cm^−1^ was likely used to assign samples to the group of vegetative plant parts. The contents of phenolic compounds (1610–1600 cm^−1^) further differentiated the stems with buds from leaves, infructescence, catkins and berries. A small but well observable shoulder at ~ 1740 cm^−1^ likely added to the separation of *D. octopetala* leaves from *Salix* stems with buds. The success of consistent accuracy to classify leaves may also be attributed to their representation by a single species and their distinctive morphology which likely created more consistent spectra for classification. The RFp classification accuracy for catkins was likely based on the distinctly increased peaks at 2920 cm^−1^, 2850 cm^−1^ and ~ 1730 cm^−1^ indicating structural lipids that might be increased in pollen and scales associated with catkins (West and Salo 1979). The contents of lipids indicated at ~ 1730 cm^−1^ likely also differentiated the leaves from stems with buds and separated permanent vegetative parts from the infructescence and catkins. Variation in the contents of phenolic compounds (1610–1600 cm^−1^) and intensities of absorbance at the OH-stretch (3490–3100 cm^−1^) allowed for differentiation of berries from infructescence. In addition, the increased absorbance of unsaturated fatty acids (3020–3000 cm^−1^) and triglyceride lipids (1745–1738 cm^−1^) was likely used to differentiate the seed containing plant parts from the remaining samples and the infructescence from the berries.

Concerning RFtp, the differentiation of berry species was likely based on an increased carbohydrate absorbance between 1035 cm^1^ and 1010 cm^−1^. Further, the increased contents of triglyceride lipids in *Vaccinium spp*. compared to *E. nigrum* berries indicated by the 3010 cm^−1^ peak attributed to unsaturated fatty acids and the band broadening in the carbonyl ester region towards ~ 1745 cm^−1^ may have been used to differentiate the berry species. Only the berries of *Vaccinium* spp. were confused with *B. nana* infructescence and *B. nana* infructescence was only confused with *Vaccinium* spp. berries, suggesting greater similarity of *B. nana* infructescence with *Vaccinium* spp. berries than *E. nigrum* berries. Concerning *Salix* stems with buds and *Betula* catkins, the lignin absorbance at 1515 cm^−1^ was likely used to differentiate *S. herbacea* from *S. phylicifolia* and *B. nana* from *B. pubescens*, yielding improved classification results compared to the RF models on raw wavenumbers. However, the differentiation within *Betula* and *Salix* species was still limited.

Based on presumed phytochemistry that distinguish plant taxa and parts, plants in crops of ptarmigan represent the range of nutrients and chemical defenses known to influence foraging of herbivores. FTIR uniquely demonstrates that ptarmigan have access to foods that allow them to balance nutritional excess and deficits of sugars predominantly provided by berries, lipids provided by reproductive parts but highest in infructescence and catkins, and protein that is likely the highest in *Salix* and *Betula* species. FTIR also uniquely demonstrates that ptarmigan face dietary trade-offs among nutrients and between nutrients and chemical defenses within taxa. While berries had a strong signal of unsaturated triglycerides (~ 3010 cm^−1^) and a distinctive signal associated with fructose ring vibrations (Max and Chapados 2007) they also had relatively low signals for protein amide I (~ 1650 cm^−1^) and II (~ 1540 cm^−1^) regions (Lin et al. 2021). In contrast, *S. herbacea* is presumed to have the highest signal for protein, but *Salix* is also defended with relatively high amounts of oxalate (~ 1620 cm^−1^, ~ 780 cm^−1^, ~ 1315 cm^−1^ (Tintner et al. 2018), tannins (~ 1720 cm^−1^ and ~ 763 cm^−1^, (Falcão and Araújo 2013), and phenolics (highest peak at ~ 1605 cm^−1^). Similar to berries, infructescence had stronger signals at ~ 3010 cm^−1^, indicative of triglycerides comprised of unsaturated fatty acids (Wang et al. 2008b) than catkins. The lipid signals in infructescence indicate higher nutritional value than catkins which had stronger methylene peaks at ~ 2920 cm^−1^ and ~ 2850 cm^−1^, indicating high hydrocarbon contents consistent with long chain fatty acid (Pasadakis et al. 2013) and distinctly increased peaks at 1731 cm^−1^, with a subsequent shoulder at 1710 cm^−1^ indicating structural lipids, possibly cutin (Mazurek et al. 2013; Heredia-Guerrero et al. 2014). Infructescence also had stronger signals of protein (~ 1650 cm^−1^ and ~ 1540 cm^−1^) and cellulose (~ 996 cm^−1^) than catkins. While relatively little information is available on the nutritional content of infructescence, catkins have relatively high energy content and may be a direct dietary source of fatty acids composition detected in grouse species (West and Meng 1966; Moss and Lough 1968). The observed reliance on catkins by ptarmigan could be attributed to presumed lower levels of phenolics (~ 1605 cm^−1^) and fiber (~ 1025 cm^−1^, ~ 996 cm^−1^) and intermediate protein levels (~ 1650 cm^−1^, ~ 1540 cm^−1^), indicating a relatively easily digestible but still nutritious food source. We recognize that the spectra identified as peaks of interest are biased to those that could be assigned to spectral features and that not all peaks could be assigned to molecular origin. In addition, we recognize that quantification of phytochemicals is required to confirm that relative peak heights represent relative concentrations of phytochemicals. Despite unknown concentrations, our identified peaks of interest can offer qualitative insights into plant physiology and interactions with herbivores. Variation in absorbance of specific peaks of interest can serve as biomarkers of relative investment in presumed phytochemicals that indicate the age of ephemeral plant parts (Boege and Marquis 2005) including the ripeness of berries (Dai et al. 2016). The spatial and temporal variation in composition or concentration of phytochemicals may explain classification accuracy of these parts. Importantly, we identified several distinctive spectral patterns, defined by specific peaks or shoulders of increased band heights, that can predict the intake of specific nutrients in future analyses based on fecal pellets. For example, the presence of a ~ 3010 cm^−1^ peak and a shoulder at ~ 1745 cm^−1^ detected in spectra of fecal pellets would indicate the intake of unsaturated fatty acids and triglycerides from infructescence or berries and berries could be further confirmed with the detection of peaks indicating fructose at ~ 818 cm^−1^ and ~ 774 cm^−1^. Increased band heights at ~ 1720 cm^−1^ and ~ 763 cm^−1^, detected in the spectra of fecal pellets could indicate the intake of tannins from *Salix*. However, the detection of these fingerprints will strongly depend on the degree of digestion or metabolization of the concerned compounds.

In conclusion, this study underscores the potential of FTIR for advancing plant and ecological research, particularly in the context of plant-herbivore interactions. Future research should prioritize expanding the spectral reference libraries to include more replicates of samples to control for phytochemical variation associated with genetic and environment conditions (Cavender-Bares et al. 2025; White et al. 2025). Future research should also include a more comprehensive range of plant species and plant parts guided by the foraging ecology of herbivores across space and time The validation of FTIR to classify food plant taxa and parts from spectral signals will accelerate future research which can use the spectra to quantify presumed phytochemicals (e.g., lipids, protein, fructose, oxalates, tannins, Table 1). Connecting spectral signals to phytochemical concentrations will enhance the taxonomic and anatomical resolution of FTIR, enabling more precise identification of plant taxa and parts consumed by herbivores. Additionally, further exploration of the FTIR method’s ability to detect subtle differences in the composition and concentration of phytochemicals could provide deeper insights into spatial and temporal variation in the primary and secondary metabolites that influence herbivore foraging ecology.

While FTIR has shown promise in distinguishing plant parts and taxa based on their spectral signatures, it is important to address its limitations in identifying plant items that may share chemical profiles. There are many spectral features that can represent phytochemicals that drive foraging behavior of herbivores. By targeting peaks of interest that where distinct among taxa or parts, we may have excluded spectral regions representing phytochemicals that are shared across all plants that influence diet selection by herbivores. FTIR can be used to investigate what combinations of similar and dissimilar foods are selected by herbivores, potentially offering new perspectives on nutritional balancing of lipids, protein, and carbohydrates (Felton et al. 2021). Moreover, applying FTIR to non-invasively collected fecal samples is the critical next step, as it will allow researchers to study plant selection repeatedly in free-ranging herbivores without direct interference. By focusing on the phytochemical traits as drivers of plant selection by herbivores, FTIR can become a cornerstone method for understanding the complex relationships between plants and herbivores, with significant implications for plant and wildlife ecology and conservation.

## Supplementary Information

Below is the link to the electronic supplementary material.

Supplementary Material 1 (PDF 467 KB)

## Data Availability

Data associated with this project is available through Github at https://github.com/zohmannM/FTIR-plant-items and at 10.5281/zenodo.19467702.
