# Supplementary material for: The Use of FTIR Spectra for Classifying Plant Items in a Vertebrate Herbivore’s Diet
Source: J Chem Ecol. 2026 May 11;52(3):41. doi: 10.1007/s10886-026-01716-4 (PMC13158255; doi:10.1007/s10886-026-01716-4)
Supplement: Supplementary file 1 — Supplementary Material 1 (PDF 467 KB) [file 10886_2026_1716_MOESM1_ESM.pdf]

Supplementary Information

JOURNAL OF CHEMICAL ECOLOGY

THE USE OF FTIR SPECTRA FOR CLASSIFYING PLANT ITEMS IN A VERTEBRATE HERBIVORE`S DIET

MARCEL SCHÄFER[1†], MARGIT ZOHMANN-NEUBERGER[1*†], JENNIFER SORENSEN FORBEY[2], JOHANNES TINTNER-OLIFIERS[3,4], ANGELIKA HROMATKA[5], ERICH INSELSBACHER[5], CHLOÉ DÉPRÉ[6,7], ÓLAFUR KARL NIELSEN[6††], URSULA NOPP-MAYR[1††]

*Corresponding author: margit.zohmann@boku.ac.at

[1] *BOKU University, Institute of Wildlife Biology and Game Management, Department of Ecosystem Management, Climate and Biodiversity, Gregor-Mendel-Strasse 33, 1180 Vienna, Austria*

[2] *Boise State University, Idaho, Department of Biological Sciences, 1910 University Drive, Boise, Idaho 83725-1515, USA*

[3] *BOKU University, Institute of Statistics, Department of Natural Sciences and Sustainable Resources, Peter Jordan-Strasse 82, 1190 Vienna, Austria*

[4] *Ernst & Young denkstatt GmbH, Hietzinger Hauptstrasse 28, 1130 Vienna, Austria*

[5] *BOKU University, Institute of Soil Research, Department of Ecosystem Management, Climate and Biodiversity, Peter Jordan-Strasse 82, 1190 Vienna, Austria*

[6] *Natural Science Institute of Iceland, Smiðjuvellir 28, 300 Akranes, Iceland*

[7] *LPO AuRA Drôme-Ardèche, 18 Pl. Genissieu, 26120 Chabeuil, France*

[†] equally contributing first authors

[††] equally contributing senior authors

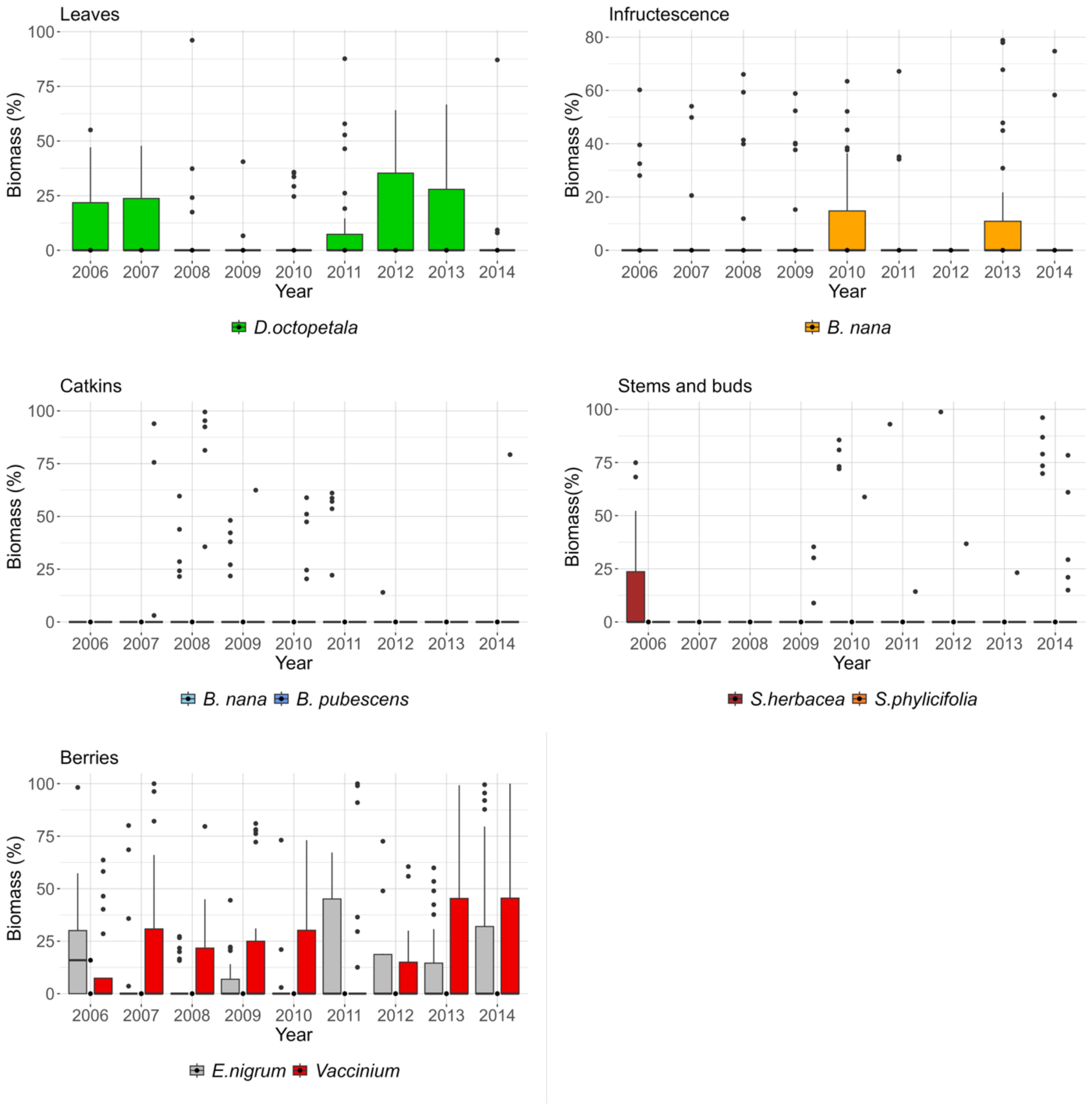

Figure S1 Relative proportion (in % of total dry mass) of the seven main plant taxa in the crops of all sampled rock ptarmigan individuals in different years, over a period of 9 years (2006-2014). (*B.nana* = *Betula nana*, *B.pubescens* = *Betula pubescens*, *D.octopetala* = *Dryas octopetala*, *E.nigrum* = *Empetrum nigrum*, S.herbacea = *Salix herbacea*, S.phylicifolia = *Salix phylicifolia*, *Vaccinium* = *Vaccinium* sp.)

Table S1 Assignment of band maxima to plausible origin according to the literature including wavenumber range listed there. Wavenumber range 3306 – 1462 cm^{-1}

**Observed wavenumber range (cm^{-1})**	**Plausible origin**	**Literature wavenumber range (cm^{-1})**	**Reference**
3600 - 3500	OH-stretching of phenolic groups in lignins<br><br>SiO-OH (H-bonded)	3568–3577<br>3620<br>3540–3500	Poletto et al. (2014), Vârban et al. (2021)<br>Volkov et al. (2021)<br>Proskurnin et al. (2023)
3304-3306	Valence vibrations of OH-groups	3400–3000	Schwanninger et al. (2004)
3016-3011	=C-H stretching of cis-alkenes in unsaturated fatty acids	3030–2990	Wang et al. (2008)
2957	Asymmetric C-H stretching of aliphatic methyl (CH_3) groups	2962	Belfer et al. (2000)
2920-2919	Asymmetric C-H stretching of aliphatic methylene (CH_2) groups	2920	Smidt et al. (2008)
2874	Symmetric C-H stretching of aliphatic CH_3 groups	2875	Belfer et al. (2000)
2851-2850	Symmetric C-H stretching of aliphatic CH_2 groups	2850	Smidt et al. (2008)
1738 -1732	C=O stretching in unconjugated ketones, carbonyl and ester groups	1738–1709	Schwanninger et al. (2004)
1720	C=O stretching vibrations in hydrolysable tannins	1731-1704	Falcão and Araújo (2013)
1650 -1605	Protein Amide I: Mainly C=O vibrational stretching	1644	Bartošová et al. (2015)
1620	Asymmetric COO^- stretching in oxalate	1622<br>1620	Piro et al. (2018)<br>Tintner et al. (2018)
1615 - 1605	C-C aromatic stretching	1615-1608<br>1606	Falcão and Araújo (2013)<br>Heredia-Guerrero et al. (2014)
1544 - 1538	Protein Amide II: Mainly N-H bending vibrations	1580-1540<br>1538	Smidt et al. (2008)<br>Bartošová et al. (2015)
1518-1512	Aromatic skeletal vibration of lignins	1515–1505	Schwanninger et al. (2004)
1462	C-H bending of hemicellulose<br>CH_2 scissoring in waxes<br>CH_2 deformation in lipids	1463<br>1463<br>1462	Bhagia et al. (2022)<br>Heredia-Guerrero et al. (2014)<br>Zimmermann and Kohler (2014)
1317-1313	Asymmetric COO^- stretching in oxalate<br>Symmetric C-O stretching of ester bonds in hydrolysable tannins	1320<br>1325-1317	Piro et al. (2018); Tintner et al. (2018)<br><br>Falcão and Araújo (2013)

1280	Condensed tannins	1288-1282	Falcão and Araújo (2013)
1162	Asymmetric C-O-C stretching in waxes<br>Asymmetric O-C-O stretching of glycosidic links	1167-1161<br>1160	Heredia-Guerrero et al. (2014)<br>Kacuráková et al. (2002)
1150-1147	Asymmetric C–O–C valence vibrations	1162-1125	Schwanninger et al. (2004)
1100	C-O-C stretching in cellulose and hemicelluloses<br>C-O and C-C stretching in cellulose	1104-1099<br>1103	Javier-Astete et al. (2021)<br>Szymanska-Chargot and Zdunek (2013)
1075	C-C and C-O stretching in hemicelluloses<br>C-C and C-O stretching in xyloglucan	1075<br>1071	Kacuráková et al. (2002)<br>Szymanska-Chargot and Zdunek (2013)
1052-1048	C-O stretching, mainly in cellulose	1055	Monti et al. (2013)
1031-1024	Aromatic C–H in-plane deformation<br>C-O stretching in holocellulose and lignin	1035–1030<br>1032-1021	Schwanninger et al. (2004)<br>Javier-Astete et al. (2021)
996	C-OH stretching in cellulose	997	Bhagia et al. (2022)
990-973	C-OH bending in starch	995	Warren et al. (2016)
968 - 960	C-O bending in pectins<br>OCH_3 in pectins	975<br>972	Szymanska-Chargot and Zdunek (2013)<br>Wilson et al. (2000)
832	C–H out-of-plane deformations in all positions, lignin H-units	834-832	Fahey et al. (2017)
817	C–C–H deformation vibrations in fructose	817, 818	Max and Chapados (2007), Svečnjak et al. (2017)
780	Symmetric O-C-O deformation in oxalate	781	Piro et al. (2018); Tintner et al. (2018)
776	C–C–H ring vibrations of fructose ring	778, 774	Max and Chapados (2007), Svečnjak et al. (2017)
762	Gallotannins	763-758	Falcão and Araújo (2013)
720	CH_2 rocking in phenolic compounds<br>CH_2 rocking in cutin and waxes	720<br>720	Álvarez et al. (2020)<br>Monazzah et al. (2018)

Table S2 Optimized hyperparameters for Random Forest models (raw wavenumbers) on plant taxa and plant parts, derived for the full spectrum and the fingerprint region, respectively (mtry = number of randomly selected variables at each split, ntree = number of decision trees in the Random Forest, maxnodes = maximum number of end nodes per decision tree, and nodesize = minimum number of observations per end node)

**Spectral region**	**Sample class**	**ntree**	**mtry**	**maxnode**	**nodesize**
full spectrum	plant parts	100	20	100	1
fingerprint	plant parts	50	30	50	1
full spectrum	plant species	300	30	100	1
fingerprint	plant species	200	10	50	1

Table S3 Occurrence (n_{occur}) and relative biomass (in %) of main plant taxa detected in the crops of sampled individual rock ptarmigan (*Lagopus muta* MONTIN) over a period of 9 years (2006-2014; n_{occur} = the total number of specimens, where the plant taxa was found in the crop; relative biomass = the ratio of the dry mass of a plant taxa to the total dry biomass within an individual's crop. *B. nana = Betula nana, B. pubescens = Betula pubescens, D. octopetala = Dryas octopetala, E. nigrum = Empetrum nigrum, S. herbacea = Salix herbacea, S. phylicifolia = Salix phylicifolia, Vaccinium = Vaccinium* spp. including both *V. uliginosum and V. myrtillus)*

**Plant taxa**	n_{occur}	**Relative biomass (%) within all sampled individuals**			
		mean	sd	min	max
B. nana	60	5.0	15.0	0	78.9
B. pubescens	16	2.1	11.3	0	95.3
D. octopetala	49	7.7	17.4	0	96.1
E. nigrum	50	8.9	21.5	0	99.5
S. herbacea	18	4.8	18.8	0	98.8
S. phylicifolia	11	1.7	8.9	0	78.4
Vaccinium spp.	72	16.4	29.7	0	100.0

Table S4 Occurrence (n_{occur}) and relative biomass (in %) of plant parts detected in the crops of sampled individual rock ptarmigan (*Lagopus muta* MONTIN) over a period of 9 years (2006-2014; n_{occur} = the total number of specimens, where the plant part was found in the crop; relative biomass = the ratio of the dry mass of a plant part to the total dry biomass within an individual's crop)

**Plant part**	$\mathbf{n_{occur}}$	**Relative biomass (%) within all sampled individuals**			
		mean	sd	min	max
Infructescence	40	7.3	18.0	0	78.9
Catkins	36	2.4	11.0	0	95.3
Berries	122	12.6	26.1	0	100.0
Stems with buds	29	3.3	14.7	0	98.8
Leaves	49	7.7	17.4	0	96.1

Table S5 Confusion matrix of the Random Forest model (raw wavenumbers) of plant parts, applied on the test data

**Prediction**	**Reference**				
	**Wind dispersed reproductive parts**		**Animal dispersed reproductive parts**	**Permanent vegetative parts**	
	**Infructescence**	**Catkins**	**Berries**	**Stems with buds**	**Leaves**
**Infructescence**	6	0	1	0	0
**Catkins**	0	8	0	0	0
**Berries**	2	0	28	0	0
**Stems with buds**	0	0	0	7	0
**Leaves**	0	0	0	0	10

Table S6. Statistics by class for the Random Forest model (raw wavenumbers) of plant parts. Values in parentheses display "total sample size/sample size used for model prediction"

	**Wind dispersed reproductive parts**		**Animal dispersed reproductive parts**	**Permanent vegetative parts**	
	**Infructescence** (40/8)	**Catkins** (36/8)	**Berries** (142/29)	**Stems with buds** (29/6)	**Leaves** (49/10)
**Sensitivity**	0.750	1.000	0.966	1.000	1.000
**Specificity**	0.981	1.000	0.939	1.000	1.000
**Pos Pred Value**	0.857	1.000	0.933	1.000	1.000
**Neg Pred Value**	0.964	1.000	0.969	1.000	1.000
**Prevalence**	0.129	0.129	0.468	0.113	0.161
**Detection Rate**	0.097	0.129	0.452	0.113	0.161
**Detection Prevalence**	0.113	0.129	0.484	0.113	0.161
**Balanced Accuracy**	0.866	1.000	0.953	1.000	1.000

Table S7 Variable importance in the Random Forest model (raw wavenumbers) for plant parts. Prevalence = number of occurrences among the 30 most important variables as indicated by the Random Forest model

**Wavenumber range (cm^{-1})**	**Prevalence**	**Mean Decrease in Accuracy**	**Presumed molecular origin (Table 1, Table S1)**
3585 - 3545	15	2.7 - 2.0	OH-stretch (lignin), SiO-H (silica)
2936 - 2912	12	2.3 - 1.9	Asymmetric stretching of methylene (CH_2) groups (lipids)
1607	1	2.5	C-C aromatic vibration (phenolics)
3037	1	2.2	No origin determined
1730	1	2	C=O stretching of ester groups (lipids)

Table S8 Confusion matrix of the Random Forest model (raw wavenumbers) of plant taxa and plant parts, applied on the test data. *B.n.* IFR= *Betula nana* infructescence*, B.n.* C = *Betula nana* catkins, *B.p.* C = *Betula pubescens* catkins*, E.n.* B = *Empetrum nigrum* berries, *V.*spp. B = *Vaccinium* spp. berries, *S.h.* Sb = *Salix herbacea* stems with buds, *S.p.* Sb = *Salix phylicifolia* stems/buds, *D.o.* L = *Dryas octopetala* leaves

**Prediction**	**Reference**							
	***B.n.* IFR**	***B.n.* C**	***B.p.* C**	***E.n.* B**	***V.*spp. B**	***S.h.* Sb**	***S.p.* Sb**	***D.o.* L**
***B.n.* IFR**	6	0	0	1	1	0	0	0
***B.n.* C**	0	4	2	0	0	0	1	0
***B.p.* C**	0	0	2	0	0	0	0	0
***E.n.* B**	0	0	0	11	1	0	0	0
***V.*spp. B**	2	0	0	2	13	0	0	0
***S.h.* Sb**	0	0	0	0	0	4	1	0
***S.p.* Sb**	0	0	0	0	0	0	1	0
***D.o.* L**	0	0	0	0	0	0	0	10

Table S9 Statistics by class for the Random Forest model (raw wavenumbers) of plant taxa and plant parts. Values in parentheses display “total sample size/sample size used for model prediction”. *B.n.* IFR= *Betula nana* infructescence*, B.n.* C = *Betula nana* catkins, *B.p.* C = *Betula pubescens* catkins*, E.n.* B = *Empetrum nigrum* berries, *V.*spp. B = *Vaccinium* spp. berries, *S.h.* Sb = *Salix herbacea* stems with buds, *S.p.* Sb = *Salix phylicifolia* stems with buds, *D.o.* L = *Dryas octopetala* leaves

	***B.n.* IFR** (40/8)	***B.n.* C** (20/4)	***B.p.* C** (16/4)	***E.n.* B** (67/14)	***V.spp.* B** (75/15)	***S.h.* Sb** (17/4)	***S.p.* Sb** (12/3)	***D.o.* L** (49/10)
**Sensitivity**	0.750	1.000	0.500	0.786	0.867	1.000	0.333	1.000
**Specificity**	0.963	0.948	1.000	0.979	0.915	0.983	1.000	1.000
**Pos Pred Value**	0.750	0.571	1.000	0.917	0.765	0.800	1.000	1.000
**Neg Pred Value**	0.963	1.000	0.967	0.940	0.956	1.000	0.967	1.000
**Prevalence**	0.129	0.065	0.065	0.226	0.242	0.065	0.048	0.161
**Detection Rate**	0.097	0.065	0.032	0.177	0.210	0.065	0.016	0.161
**Detection Prevalence**	0.129	0.113	0.032	0.194	0.274	0.081	0.016	0.161
**Balanced Accuracy**	0.856	0.974	0.750	0.882	0.891	0.991	0.667	1.000

Table S10 Variable importance in the Random Forest model (raw wavenumbers) for plant taxa and plant parts. Prevalence = number of occurrences among the 30 most important variables as indicated by the Random Forest model

Wavenumber range (cm^{-1})	Prevalence	Mean Decrease in Accuracy	Presumed molecular origin (Table 1, Table S1)
1134 - 1123	16	4 - 3.6	No origin determined
1751 - 1741	12	4.0 - 3.6	C=O stretching in triglycerides (lipids)
3547 – 3533	2	3.8 - 3.6	OH-stretch (lignin), SiO-H (silica)

Table S11 Defined wavenumber ranges for feature aggregation to be used in the Random Forest models on aggregated bands. References for the attributed molecular origins are given in Table 1 and Table S1

Band aggregate (cm^{-1})	Presumed molecular origin (Table 1, Table S1)	Upper limit (cm^{-1})	Lower limit (cm^{-1})
3600-3500	OH (silica and lignins)	3600	3500
3490-3100	OH (carbohydrates)	3490	3100
3020-3000	Unsaturated fatty acids	3020	3000
2930-2910	Asymmetric CH2	2930	2910
2860-2840	Symmetric CH2	2860	2840
1745-1738	Triglycerides lipids	1745	1738
1733-1730	Cutin (~1730)	1733	1730
1711-1708	Cutin (~1710)	1711	1708
1722-1716	Hydrolysable tannins (~1720)	1722	1716
1670-1625	Protein Amide I	1670	1625
1580-1520	Protein Amide II	1580	1520
1620-1618	Oxalate (1620)	1620	1618
1610-1600	Phenolics (~1607)	1610	1600
1515-1500	Lignin	1515	1500
1140-1128	Carbohydrate Fingerprint	1140	1128
1108-1098	Carbohydrate Fingerprint	1108	1098
1084-1070	Carbohydrate Fingerprint	1084	1070
1058-1046	Carbohydrate Fingerprint	1058	1046
1035-1010	Carbohydrate Fingerprint	1035	1010
1000-990	Cellulose	1000	990
975-960	Pectin	975	960
820-815	Fructose (~818)	820	815
779-773	Fructose (~774)	779	773
782-778	Oxalate (780)	782	778
1288-1282	Condensed tannins (~1280)	1288	1282
763-758	Hydrolysable tannins (~763)	763	758

Table S12 Optimized hyperparameters for Random Forest models (aggregated bands) for plant taxa and plant parts, derived for the full spectrum (mtry = number of randomly selected variables at each split, ntree = number of decision trees in the Random Forest, maxnodes = maximum number of end nodes per decision tree, and nodesize = minimum number of observations per end node)

Spectral region	Sample class	ntree	mtry	maxnodes	nodesize
full spectrum	plant parts	500	5	50	1
full spectrum	plant taxa and parts	100	5	100	1

Table S13 Confusion matrix of the Random Forest model (aggregated bands) for plant parts (RFp), applied on the test data

**Prediction**	**Reference**				
	**Wind dispersed reproductive parts**		**Animal dispersed reproductive parts**	**Permanent vegetative parts**	
	**Infructescence**	**Catkins**	**Berries**	**Stems with buds**	**Leaves**
**Infructescence**	6	0	0	0	0
**Catkins**	0	8	0	0	0
**Berries**	2	0	29	0	0
**Stems with buds**	0	0	0	6	0
**Leaves**	0	0	0	0	10

Table S14 Statistics by class of the Random Forest model (aggregated bands) for plant parts (RFp). Values in parentheses display “total sample size/sample size used for model prediction”

	**Wind dispersed reproductive parts**		**Animal dispersed reproductive parts**	**Permanent vegetative parts**	
	**Infructescence** (40/8)	**Catkins** (36/8)	**Berries** (142/29)	**Stems with buds** (29/6)	**Leaves** (49/10)
**Sensitivity**	0.750	1.000	1.000	1.000	1.000
**Specificity**	1.000	1.000	0.938	1.000	1.000
**Pos Pred Value**	1.000	1.000	0.935	1.000	1.000
**Neg Pred Value**	0.964	1.000	1.000	1.000	1.000
**Prevalence**	0.131	0.131	0.475	0.098	0.164
**Detection Rate**	0.098	0.131	0.475	0.098	0.164
**Detection Prevalence**	0.098	0.131	0.508	0.098	0.164
**Balanced Accuracy**	0.875	1.000	0.969	1.000	1.000

Table S15 Variable importance calculated from 30-fold cross-validation of the Random Forest model (aggregated bands) for plant parts (RFp), applied on the training data

Band aggregation (cm^{-1})	Mean Decrease in Accuracy	Presumed molecular origin (Table 1, Table S1)
1320-1310	22.829	Oxalate
2860-2840	20.249	Symmetric CH_2 (lipids)
3490-3100	19.239	OH-stretch (carbohydrates)
1610-1600	17.177	Phenolic compounds
2930-2910	16.425	Asymmetric CH_2 (lipids)
1733-1730	15.922	Lipids (cutin)
1745-1738	15.638	Lipids (triglycerides)
3020-3000	14.064	Unsaturated fatty acids
820-815	13.361	Fructose
1722-1716	12.499	Tannins
782-780	12.213	Oxalate
779-773	10.113	Fructose
1165-1156	9.748	Carbohydrates
1515-1500	9.676	Lignin
1035-1010	8.869	Carbohydrates
1288-1282	8.657	Tannins
975-960	8.608	Pectin
763-758	7.405	Tannins
1580-1520	7.346	Protein amide II
1058-1046	7.036	Carbohydrates
1155-1140	6.309	Carbohydrates
1670-1625	5.215	Protein amide I
3600-3500	4.253	OH-stretch (silica and lignins)
1620-1618	3.853	Oxalate
1000-990	3.681	Cellulose
1711-1708	-0.321	Lipids (cutin)

Table S16 Confusion matrix of the Random Forest model (aggregated bands) for plant taxa and plant parts (RFtp), applied on the test data. *B.n.* IFR= *Betula nana* infructescence, *B.n.* C = *Betula nana* catkins, *B.p.* C = *Betula pubescens* catkins, *E.n.* B = *Empetrum nigrum* berries, *V.*spp. B = *Vaccinium* spp. berries, *S.h.* Sb = *Salix herbacea* stems with buds, *S.p.* Sb = *Salix phylicifolia* stems/buds, *D.o.* L = *Dryas octopetala* leaves

**Prediction**	**Reference**							
	B.n. **IFR**	***B.n.*** **C**	***B.p.*** **C**	***E.n.*** **B**	***V.*****spp. B**	***S.h.*** **Sb**	***S.p.*** **Sb**	***D.o.*** **L**
B.n. **IFR**	7	0	0	0	1	0	0	0
B.n. **C**	0	3	1	0	0	0	0	0
B.p. **C**	0	1	3	0	0	0	0	0
E.n. **B**	0	0	0	12	1	0	0	0
V.**spp. B**	1	0	0	2	13	0	0	0
S.h. **Sb**	0	0	0	0	0	3	1	0
S.p. **Sb**	0	0	0	0	0	1	2	0
D.o. **L**	0	0	0	0	0	0	0	10

Table S17 Statistics by class of the Random Forest model (aggregated bands) for plant taxa and plant parts (RFtp). Values in parentheses display "total sample size/sample size used for model prediction". *B.n.* IFR= *Betula nana* infructescence, *B.n.* C = *Betula nana* catkins, *B.p.* C = *Betula pubescens* catkins, *E.n.* B = *Empetrum nigrum* berries, *V.*spp. B = *Vaccinium* spp. berries, *S.h.* Sb = *Salix herbacea* stems with buds, *S.p.* Sb = *Salix phylicifolia* stems with buds, *D.o.* L = *Dryas octopetala* leaves

	***B.n.* IFR** (40/8)	***B.n.* C** (20/4)	***B.p.* C** (16/4)	***E.n.* B** (67/14)	***V.*spp. B** (75/15)	***S.h.* Sb** (17/4)	***S.p.* Sb** (12/3)	***D.o.* L** (49/10)
**Sensitivity**	0.875	0.750	0.750	0.857	0.867	0.750	0.666	1.000
**Specificity**	0.981	0.983	0.983	0.970	0.936	0.983	0.936	1.000
**Pos Pred Value**	0.875	0.750	0.750	0.923	0.813	0.750	0.666	1.000
**Neg Pred Value**	0.981	0.983	0.983	0.959	0.957	0.983	0.983	1.000
**Prevalence**	0.129	0.065	0.065	0.226	0.242	0.065	0.048	0.163
**Detection Rate**	0.129	0.048	0.048	0.193	0.210	0.048	0.032	0.163
**Detection Prevalence**	0.129	0.065	0.065	0.210	0.258	0.065	0.048	0.163
**Balanced Accuracy**	0.928	0.866	0.866	0.918	0.901	0.866	0.825	1.000

Table S18 Variable importance calculated from 30-fold cross-validation of the Random Forest model (aggregated bands) for plant taxa and plant parts (RFtp), applied on the training data

Band aggregation (cm^{-1})	Mean Decrease in Accuracy	Presumed molecular origin (Table 1, Table S1)
1035-1010	10.1806816	Carbohydrates
3020-3000	9.1172260	Unsaturated fatty acids
1745-1738	9.1049289	Lipids (triglyceride)
1320-1310	8.5009570	Oxalate
3490-3100	8.3395595	OH-stretch (carbohydrates)
1515-1500	7.3554774	Lignin
2930-2910	7.3130654	Asymmetric CH_2 (lipids)
1733-1730	6.5876018	Lipids (cutin)
2860-2840	6.5120116	Symmetric CH_2 (lipids)
1610-1600	6.3300524	Phenolics
763-758	6.1347925	Tannins
1670-1625	6.0945416	Protein Amide I
1580-1520	6.0687859	Protein Amide II
1722-1716	5.6078133	Tannins
782-780	5.5750627	Oxalate
820-815	5.4741299	Fructose
1165-1156	4.7894110	Carbohydrates
779-773	4.4096391	Fructose
1288-1282	4.3821514	Tannins
975-960	4.2452902	Pectin
1155-1140	3.9506723	Carbohydrates
1058-1046	3.8898539	Carbohydrates
3600-3500	2.5815887	OH-stretch (silica and lignins)
1620-1618	2.2243722	Oxalate
1000-990	1.8035618	Cellulose
1711-1708	-0.1914921	Lipids (cutin)

References

Álvarez À, Yáñez J, Neira Y, Castillo-Felices R, Hinrichsen P (2020) Simple Distinction of Grapevine (*Vitis vinifera* L.) Genotypes by Direct ATR-FTIR. Food Chemistry 328:127–164. https://doi.org/10.1016/j.foodchem.2020.127164

Bartošová A, Blinová L, Gerulová K (2015) Characterisation of Polysacharides and Lipids from Selected Green Algae Species by FTIR-ATR Spectroscopy. Research Papers. Faculty of Materials Science and Technology. Slovak University of Technology in Bratislava 23:97–102. https://doi.org/10.1515/rput-2015-0011

Belfer S, Fainchtain R, Purinson Y, Kedem O (2000) Surface Characterization by FTIR-ATR Spectroscopy of Polyethersulfone Membranes - Unmodified, Modified and Protein fouled. Journal of Membrane Science 172:113–124. https://doi.org/10.1016/s0376-7388(00)00316-1

Bhagia S, Ďurkovič J, Lagaňa R, Kardošová M, Kačík F, Cernescu A, Schäfer P, Yoo CG, Ragauskas AJ (2022) Nanoscale FTIR and Mechanical Mapping of Plant Cell Walls for Understanding Biomass Deconstruction. ACS Sustainable Chemistry & Engineering 10:3016–3026. https://doi.org/10.1021/acssuschemeng.1c08163

Fahey LM, Nieuwoudt MK, Harris PJ (2017) Predicting the Cell-Wall Compositions of *Pinus radiata* (radiata pine) Wood using ATR and Transmission FTIR Spectroscopies. Cellulose 24:5275–5293. https://doi.org/10.1007/s10570-017-1506-4

Falcão L, Araújo MEM (2013) Tannins Characterization in Historic Leathers by Complementary Analytical Techniques ATR-FTIR, UV-Vis and Chemical Tests. Journal of Cultural Heritage 14:499–508. https://doi.org/10.1016/j.culher.2012.11.003

Heredia-Guerrero JA, Benítez JJ, Domínguez E, Bayer IS, Cingolani R, Athanassiou A, Heredia A (2014) Infrared and Raman Spectroscopic Features of Plant Cuticles: A Review. Frontiers in Plant Science 5:305. https://doi.org/10.3389/fpls.2014.00305

Javier-Astete R, Jimenez-Davalos J, Zolla G (2021) Determination of Hemicellulose, Cellulose, Holocellulose and Lignin Content using FTIR in *Calycophyllum spruceanum* (Benth.) K. Schum. and *Guazuma crinita* Lam. PLoS One 16. https://doi.org/10.1371/journal.pone.0256559

Kacuráková M, Smith AC, Gidley MJ, Wilson RH (2002) Molecular Interactions in Bacterial Cellulose Composites Studied by 1D FT-IR and Dynamic 2D FT-IR Spectroscopy. Carbohydrate Research 337:1145–1153. https://doi.org/10.1016/s0008-6215(02)00102-7

Max J-J, Chapados C (2007) Glucose and Fructose Hydrates in Aqueous Solution by IR Spectroscopy. J Phys Chem A 111:2679–2689. https://doi.org/10.1021/jp066882r

Monazzah M, Soleimani MJ, Tahmasebi Enferadi S, Rabiei Z (2018) Effects of Oxalic Acid and Culture Filtrate of *Sclerotinia sclerotiorum* on Metabolic Changes in Sunflower evaluated using FT-IR Spectroscopy. Journal of General Plant Pathology 84:2–11

Monti F, Dell'Anna R, Sanson, A., Fasoli, M., Pezzotti, M., Zenoni S (2013) A Multivariate Statistical Analysis Approach to Highlight Molecular Processes in Plant Cell Walls through ATR FT-IR Microspectroscopy: The Role of the α-Expansin PhEXPA1 in *Petunia hybrida.* Vibrational Spectroscopy 65:36–43

Piro OE, Echeverría GA, González-Baró AC, Baran EJ (2018) Crystal Structure and Spectroscopic Behavior of Synthetic Novgorodovaite Ca2(C2O4)Cl2·2H2O and its Twinned Triclinic Heptahydrate Analog. Physics and Chemistry of Minerals 45:185–195. https://doi.org/10.1007/s00269-017-0907-0

Poletto M, Ornaghi HL, Zattera AJ (2014) Native Cellulose: Structure, Characterization and Thermal Properties. Materials 7:6105–6119. https://doi.org/10.3390/ma7096105

Proskurnin MA, Volkov DS, Rogova OB (2023) Temperature Dependences of IR Spectral Bands of Humic Substances of Silicate-Based Soils. Agronomy 13:1740. https://doi.org/10.3390/agronomy13071740

Schwanninger M, Rodrigues JC, Pereira H, Hinterstoisser B (2004) Effects of Short-Time Vibratory Ball Milling on the Shape of FT-IR Spectra of Wood and Cellulose. Vibrational Spectroscopy 36:23–40. https://doi.org/10.1016/j.vibspec.2004.02.003

Smidt E, Meissl K, Schwanninger M, Lechner P (2008) Classification of Waste Materials using Fourier Transform Infrared Spectroscopy and Soft Independent Modeling of Class Analogy. Waste Management 28:1699–1710. https://doi.org/10.1016/j.wasman.2007.08.003

Svečnjak L, Prđun S, Rogina J, Bubalo D, Jerković I (2017) Characterization of Satsuma mandarin (*Citrus unshiu* Marc.) Nectar-To-Honey Transformation Pathway Using FTIR-ATR Spectroscopy. Food Chemistry 232:286–294. https://doi.org/10.1016/j.foodchem.2017.03.159.

Szymanska-Chargot M, Zdunek A (2013) Use of FT-IR Spectra and PCA to the Bulk Characterization of Cell Wall Residues of Fruits and Vegetables Along a Fraction Process. Food Biophysics 8:29–42. https://doi.org/10.1007/s11483-012-9279-7

Tintner J, Smidt E, Aumüller C, Martin P, Ottner F, Wriessnig K, Reschreiter H (2018) Taphonomy of Prehistoric Bark in a Salt Environment at the Archaeological Site in Hallstatt, Upper Austria – An Analytical Approach Based on FTIR Spectroscopy. Vibrational Spectroscopy 97:39–43. https://doi.org/10.1016/j.vibspec.2018.05.006

Vârban R, Crișan I, Vârban D, Ona A, Olar L, Stoie A, Ștefan R (2021) Comparative FT-IR Prospecting for Cellulose in Stems of Some Fiber Plants: Flax, Velvet Leaf, Hemp and Jute. Applied Sciences 11:8570. https://doi.org/10.3390/app11188570

Volkov D, Rogova O, Proskurnin M (2021) Organic Matter and Mineral Composition of Silicate Soils: FTIR Comparison Study by Photoacoustic, Diffuse Reflectance, and Attenuated Total Reflection Modalities. Agronomy 11:1879. https://doi.org/10.3390/agronomy11091879

Wang Y, Wang Q, Artz WE, Padua GW (2008) Fourier Transform Infrared Spectra of Drying Oils Treated by Irradiation. J Agric Food Chem 56:3043–3048. https://doi.org/10.1021/jf073545m

Warren FJ, Gidley MJ, Flanagan BM (2016) Infrared Spectroscopy as a Tool to Characterise Starch Ordered Structure -A Joint FTIR-ATR, NMR, XRD and DSC Study. Carbohydrate Polymers 139:35–42. https://doi.org/10.1016/j.carbpol.2015.11.066

Wilson RH, Smith AC, Kâcuráková M, Saunders PK, Wellner N, Waldron KW (2000) The Mechanical Properties and Molecular Dynamics of Plant Cell Wall Polysaccharides Studied by Fourier-Transform Infrared Spectroscopy. Plant Physiology 124:397–405. https://doi.org/10.1104/pp.124.1.397

Zimmermann B, Kohler A (2014) Infrared spectroscopy of pollen identifies plant species and genus as well as environmental conditions. PLoS One 9. https://doi.org/10.1371/journal.pone.0095417
